# Early warning of serotype replacement, spatial risk redistribution, and shifting clinical phenotypes: Insights from the 2023–2025 dengue outbreak in Meta, Colombia

**DOI:** 10.1371/journal.pntd.0014733

**Published:** 2026-09-25

**Authors:** Liliana Marcela Montilla Rodríguez, Cesar García-Balaguera, Sandra Campo Palacio, David Bonilla Montes, Yuri Daniela Cadena Díaz, Paula Alejandra Mosquera Tocora, Norma Cristina Pavas Escobar, Camilo Andrade Carreño, Elsa Victoria Andrade Miranda, Marina Sthella Gonzalez Robayo

**Affiliations:** 1 Faculty of Medicine, Universidad Cooperativa de Colombia, Villavicencio, Colombia; 2 GRIVI, Villavicencio, Colombia; 3 Laboratorio de Salud Pública, GISSME, Secretaría de Salud del Meta, Villavicencio, Colombia; Jigjiga University, ETHIOPIA

## Abstract

**Background:**

Dengue remains a major public health challenge in hyperendemic regions, where shifts in serotype circulation, spatial transmission patterns, and clinical presentation can significantly influence outbreak dynamics. Colombia has experienced intense dengue activity in recent years; however, integrated analyses combining virological, spatial, and clinical data remain limited.

**Methods:**

A retrospective longitudinal observational study was conducted using routinely collected dengue surveillance data from the Department of Meta, Colombia, covering the period from January 2023 to September 2025. Confirmed dengue cases with viral isolation and serotype identification were included. Temporal trends, serotype replacement dynamics, municipal-level spatial redistribution patterns, and clinical characteristics were analyzed using laboratory-confirmed dengue cases. Multivariable logistic regression models were used to identify factors associated with dengue positivity overall and by serotype, while interannual variation was assessed to evaluate changes in transmission intensity and the spatial redistribution of dengue transmission risk.

**Results:**

Marked serotype replacement was observed during the study period. DENV-2 predominated during the 2023 peak, followed by a decline and a sustained increase in DENV-3 and DENV-4 by 2025, while DENV-1 disappeared from recent records. Spatial analysis showed declining diagnostic positivity in historically hyperendemic municipalities and significant interannual increases in intermediate municipalities, indicating an epidemiological rebound outside major urban centers. Classical dengue symptoms, including headache, myalgia, and rash, were inversely associated with virological confirmation, whereas nonspecific neurological manifestations, such as somnolence and irritability, emerged as independent predictors. Severe thrombocytopenia remained strongly associated with infection, particularly in DENV-2 cases.

**Conclusions:**

The dengue outbreak in Meta (2023–2025) was characterized by dynamic serotype replacement, spatial redistribution of transmission risk, and changing clinical presentations. The rapid emergence of DENV-4 in 2025 serves as an early warning of serotype replacement and underscores the need to update clinical screening protocols to include nonspecific neurological manifestations. Strengthening decentralized laboratory networks and closing surveillance gaps in low-endemicity areas are strategic priorities to improve early detection and outbreak control in hyperendemic regions.

## Author summary

Dengue is one of the most important mosquito-borne diseases worldwide and remains a major public health challenge in tropical regions. In Colombia, recurrent outbreaks are driven by the circulation of different dengue virus serotypes, which can influence disease transmission patterns and clinical presentation. We analyzed routine surveillance data from the Department of Meta, Colombia, collected between 2023 and September 2025 to investigate changes in serotype circulation, geographic distribution, and clinical characteristics of dengue cases. We found evidence of a rapid replacement of dengue virus serotypes during the outbreak period. DENV-2 predominated during the major epidemic peak in 2023, but its circulation progressively declined while DENV-3 increased and DENV-4 showed a marked rise beginning in 2024 and continuing through 2025, despite its historically limited circulation in the Department of Meta. Spatial analyses also revealed a redistribution of transmission risk from traditionally high-burden municipalities toward intermediate-transmission areas. In addition, some nonspecific neurological manifestations, including somnolence and irritability, emerged as important predictors of laboratory-confirmed dengue infection. These findings provide an early warning of changing dengue epidemiology in Colombia and highlight the importance of strengthening virological surveillance, improving laboratory capacity, and updating clinical screening strategies to support timely outbreak detection and public health response.

## 1. Introduction

Dengue virus infection is one of the foremost public health priorities and challenges in tropical and subtropical regions, owing to its high incidence, broad geographic expansion, and complex transmission dynamics. Over recent decades, its incidence has shown a sustained upward trend, driven by factors such as unplanned urban growth, climatic variability, and the vector’s ability to adapt to new environments. Globally, the World Health Organization (WHO) reports that dengue transmission occurs in nearly 90 countries, many of which lack robust detection and reporting systems, suggesting that the true burden of the disease may be significantly underestimated [1].

According to World Health Organization (WHO) estimates, approximately 390 million dengue infections occur each year, of which around 96 million presents with clinical. This magnitude positions dengue as the most impactful arboviral disease worldwide. In the Region of the Americas, particularly between 2023 and 2024, an epidemic pattern was observed, characterized by a sustained increase in case incidence, reflecting intense viral circulation and the reemergence of multiple serotypes in endemic countries [2]. Colombia reported 175,962 cases between epidemiological week (EW) 1 and EW 23 of 2024, representing a 352% increase compared with the five-year average for the same period in the country. The cumulative incidence rates up to EW 23 was 343 cases per 100,000 inhabitants, of which 1,592 cases (0.90%) were classified as severe. A total of 81 deaths were recorded, corresponding to a case-fatality rate of 0.046% [3,4].

In Colombia, dengue is an endemic disease characterized by cyclical epidemic peaks that often coincide with periods of increased rainfall and with the population growth of the *Aedes* vector. Over the past decade, the Orinoquía region—particularly the department of Meta—has shown a sustained rise in the incidence and frequency of outbreaks, associated with the simultaneous circulation of multiple viral serotypes [5–9]. This situation reflects the convergence of ecological, virological, and socioeconomic factors that modulate transmission and complicate both vector control strategies and timely diagnosis. According to the National Institute of Health (INS), a new epidemic phase of dengue began in Colombia in 2023, consistent with the pattern observed in several countries across the Americas. During that year, the incidence reached 387.2 cases per 100,000 inhabitants, a figure higher than that reported in other recent epidemic periods. The national case-fatality rate was 0.09%, slightly below the target established in the Ten-Year Public Health Plan 2022–2031 (<0.10%). However, some territories exceeded this threshold, including Amazonas, Antioquia, Bolívar, Chocó, La Guajira, Magdalena, Nariño, Norte de Santander, and Sucre. In addition, departments such as Amazonas, Arauca, Bolívar, Cauca, Chocó, Cundinamarca, Guaviare, La Guajira, Meta, Nariño, Norte de Santander, and Sucre experienced outbreak conditions during more than 90% of the epidemiological weeks of the year [4,10].

This arboviral disease, transmitted primarily by *Aedes aegypti* and *Aedes albopictus*, maintains persistent circulation in Latin America, where the coexistence of the four viral serotypes (DENV-1 to DENV-4) and the emergence of new genotypes have facilitated the occurrence of epidemic outbreaks with unpredictable behavior and, in some cases, severe forms of the disease. Since 2009, the WHO has classified dengue according to clinical complexity into three categories: dengue without warning signs, dengue with warning signs, and severe dengue—a category that includes dengue shock syndrome and complications such as myocarditis, encephalitis, and hepatitis, all associated with an increased risk of mortality [1,9]. Typical clinical manifestations in uncomplicated phases include sudden-onset fever, severe headache, retro-orbital pain, myalgia, arthralgia, and rash, symptoms that often vary according to the patient’s age [4,11,12].

In this context, identifying recent changes in the genetic composition of the circulating serotypes in the country becomes particularly important. Recently, Martínez et al. reported the emergence of the Cosmopolitan genotype of serotype 2 (DENV-2) in Colombia, a finding of substantial epidemiological relevance, as this lineage has demonstrated a high capacity for expansion in Asia, Africa, and the Pacific. Its presence in endemic regions may significantly alter local transmission dynamics [5]. The documentation of this genotype circulating in Colombia provides evidence of a recent introduction that may influence the magnitude, frequency, and intensity of outbreaks, as well as the geographic distribution of serotype 2. This underscores the need to strengthen surveillance systems to anticipate potential changes in clinical severity and in the epidemiological behavior of dengue at the national level. The introduction of highly adaptable lineages, such as the Cosmopolitan genotype of DENV-2, raises the question of how these new variants interact with pre-existing population immunity to facilitate serotype replacement [13]. In this context, genomic and virological surveillance becomes critical to determine whether the recent dominance of DENV-4 in Meta reflects a vacant-niche dynamic or an intrinsic biological advantage over locally circulating DENV-2 variants [14,15].

Despite the existence of entomological and virological surveillance systems, significant gaps remain in the characterization of clinical, epidemiological, and virological predictors associated with dengue virus infection in local contexts [16,17]. Likewise, information on the distribution of circulating serotypes and their possible relationship with disease severity remains limited [18,19]. This lack of knowledge hinders the early detection of changes in transmission patterns and the implementation of effective control interventions [14]. In this regard, the reintroduction of serotype 3 (DENV-3), documented in several countries in the region during the 2023–2024 period, could increase the risk of severe clinical presentations in populations previously exposed to other serotypes [2].

The present study aimed to describe and analyze the clinical, epidemiological, and virological characteristics—including serotype distribution—of dengue virus infection cases reported in the department of Meta during the 2023–2025 period. Through a retrospective longitudinal observational design based on routinely collected surveillance data and the diagnostic database of the Public Health Laboratory of Meta, the principal predictors associated with dengue virus positivity and serotype distribution were evaluated over time. The findings of this study aim to provide evidence for a more precise understanding of the clinical and epidemiological behavior of dengue in endemic regions of Colombia and other countries with similar epidemiological, ecological, and sociodemographic characteristics, contributing to the strengthening of early warning systems, the optimization of clinical case management, and the guidance of public health decision-making at both regional and international levels.

## 2. Results

### 2.1. Sociodemographic and clinical characteristics of serotyped dengue cases

#### 2.1.1. Sociodemographic characteristics, clinical classification, and case management.

Table 1a presents the sociodemographic, diagnostic, clinical management characteristics and temporal distribution of dengue cases reported in the Department of Meta during the outbreak that occurred between 2023 and September 2025. The annual distribution of serotyped cases revealed a higher concentration in 2023, accounting for 53.2% (n = 1,782) of records, followed by 2025 with 24.9% (n = 834) and 2024 with 21.9% (n = 733). Regarding sex distribution, there was a slight predominance of males (56.5%; n = 1,892) compared to females (43.5%; n = 1,457). With respect to health insurance affiliation, 55.1% of patients were enrolled in the subsidized scheme (state-funded), while 44.9% belonged to the contributory scheme (funded by employees and employers). According to the final clinical classification, most cases were categorized as dengue with warning signs (57.3%), followed by dengue without warning signs (34.9%), severe dengue (1.0%), and unclassified cases (6.8%) due to incomplete clinical information. Regarding clinical management, most patients required general hospitalization (60%), while 23.1% were treated on an outpatient basis, 15.8% remained under observation, and only 1% required care in the intensive care unit (ICU).

**Table 1:** Characteristics of the 2023–2025 Dengue Outbreak in Meta, Colombia.

a. Sociodemographic and clinical management characteristics of confirmed dengue cases.						
Variable				Dengue-positive cases reported n =3717	Confirmed dengue virus serotyping n= 3349	
				n (%)	n (%)	
Year	2023			2058(55.4)	1782(53.2)	
	2024			737(19.8)	733(21.9)	
	2025			922(24.8)	834(24.9)	
Sex	Female			1632(43.9)	1457(43.5)	
	Male			2085(56.1)	1892(56.5)	
Health insurance type	Contributory scheme (funded by employee/employer)			1665(44.8)	1504(44.9)	
	Subsidized scheme (funded by the State)			2052(55.2)	1845(55.1)	
Type of analysis performed	AgNS1- ELISA			91(2.4)	-	
	IgM ELISA			277(7.5)	-	
	RT-PCR Nucleic Acid Detection			3349(90.1)	3349(100)	
Final dengue classification	Severe dengue			38(1)	33(1)	
	Dengue with warning signs			2204(59.3)	1919(57.3)	
	Dengue without warning signs			1210(32.6)	1169(34.9)	
	Unclassified			265(7.1)	228(6.8)	
Clinical management	Outpatient			802(21.6)	775(23.1)	
	General hospitalization			2300(61.9)	2011(60)	
	Observation			564(15.2)	530(15.8)	
	ICU			51(1.4)	33(1)	
**b. Clinical characteristics of cases with confirmed dengue virus serotyping (n=3349)**						
Variable		n (%)	Variable			n (%)
Fever	No	252(7.5)	Severe mucosal bleeding		No	3259(97.3)
	Yes	3097(92.5)			Yes	90/2.7)
Headache	No	882(26.3)	Hypothermia		No	3336(99.6)
	Yes	2467(73.7)			Yes	13(0.4)
Retro-orbital pain	No	1815(54.2)	Hematocrit increase		No	3229(96.4)
	Yes	1534(45.8)			Yes	120(3.6)
Myalgia	No	624(18.6)	Platelet count <100,000		No	2242(66.9)
	Yes	2725(81.4)			Yes	1107(33.1)
Arthralgia	No	632(18.9)	Fluid accumulation		No	3322(99.2)
	Yes	2717(81.1)			Yes	27(0.8)
Skin rash	No	2764(82.5)	Severe plasma leakage		No	3339(99.7)
	Yes	585(17.5)			Yes	10(0.3)
Abdominal pain	No	2370(70.8)	Bleeding with hemodynamic compromise		No	3341(99.8)
	Yes	979(29.2)			Yes	8(0.2)
Diarrhea	No	2832(84.6)	Dengue shock		No	3343(99.8)
	Yes	517(15.4)			Yes	6(0.2)
Somnolence / Irritability	No	3266(97.5)	Severe organ damage		No	3337(99.6)
	Yes	83(2.5)			Yes	2(0.4)
Hypotension	No	3320(99.1)	Emesis		No	2661(79.5)
	Yes	29(0.9)			Yes	688(20.5)
Hepatomegaly	No	3326(99.3)				
	Yes	23(0.7)				

Table 1a. Distribution of dengue cases according to year of notification, sex, type of health insurance, diagnostic method, final clinical classification, and clinical management. Data are presented for the total cohort of suspected cases (n = 3,717) and for the subset of cases with confirmed dengue virus serotyping by RT-PCR Nucleic Acid Detection (n = 3,349). Values are expressed as absolute frequencies and percentages.

Table 1b. Clinical manifestations and severity markers of dengue cases with laboratory-confirmed serotyping during the 2023–September 2025 outbreak in the Department of Meta, Colombia (n = 3,349). Values are expressed as n (%). Clinical features, warning signs, and severe dengue criteria were classified according to WHO guidelines, including hematological alterations, plasma leakage, mucosal bleeding, hemodynamic compromise, dengue shock, and severe organ involvement.

#### 2.1.2. Clinical manifestations among cases with confirmed dengue virus serotyping.

Of the total 3,717 samples analyzed during the dengue outbreak that occurred between 2023 and September 2025, this analysis included 3,349 cases with confirmed dengue virus serotyping, processed through RT-PCR Nucleic Acid Detection, thus constituting the cohort with complete virological information. **Table 1b** summarizes the main clinical characteristics of this population. The most frequent clinical manifestations included fever (92.5%), headache (73.7%), myalgia (81.4%), and arthralgia (81.1%), symptoms characteristic of the acute febrile phase. Additionally, emesis (20.5%) and abdominal pain (29.2%) were reported, both considered warning signs according to the World Health Organization (WHO) guidelines. Severe hemorrhagic manifestations were uncommon, with severe mucosal bleeding observed in 2.7% and bleeding with hemodynamic compromise in only 0.2% of cases. Similarly, hypotension (0.9%), hepatomegaly (0.7%), and severe plasma leakage (0.3%) were infrequent findings.

### 2.2. Temporal dynamics and serotype circulation during the 2023–2025 dengue outbreak

#### 2.2.1. Temporal dynamics of dengue cases and diagnostic positivity (2023–2025).

**Fig 1:**
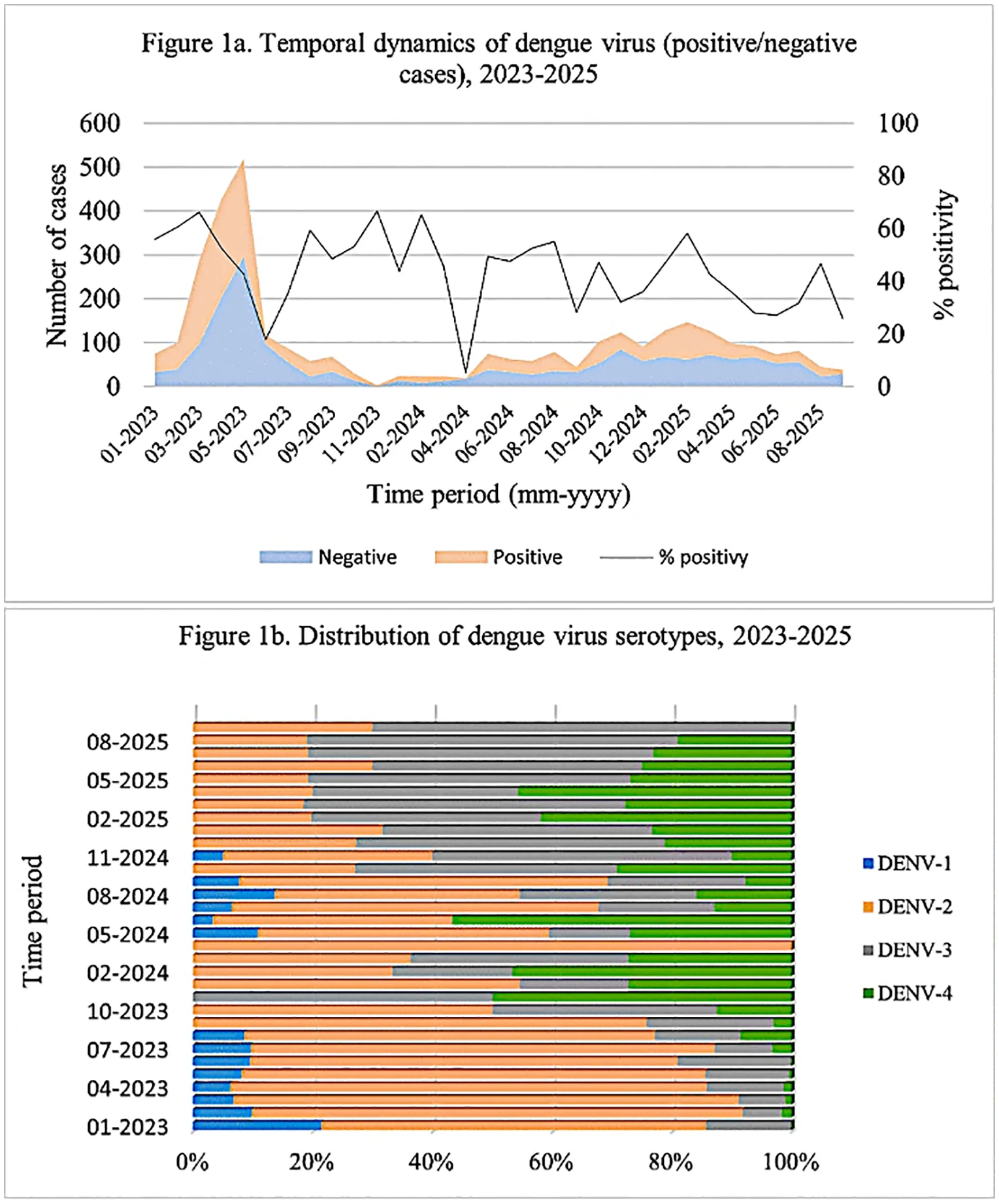
a. Temporal dynamics of dengue diagnostic results and positivity, 2023–2025. The figure displays the absolute number of positive and negative samples and the monthly diagnostic positivity, showing an epidemic peak in the first half of 2023, followed by a stabilization phase with seasonal fluctuations throughout 2024 and 2025. Diagnostic positivity was calculated as the proportion of positive samples relative to the total number of samples processed each month (positive and negative samples).b. Distribution of dengue virus serotypes, 2023-2025. An initial predominance of DENV-2 is observed in 2023, followed by a progressive increase in DENV-3 from 2024 onward and the emergence of DENV-4 as the predominant serotype in 2025, in agreement with the data summarized in Table 2.

Of the 3,717 clinical samples, 51.2% (n = 1,904) tested positive and 48.8% (n = 1,813) tested negative using routine diagnostic tests. When considering exclusively the samples with confirmed dengue virus serotyping (n = 3,349), the overall dengue virus positivity rate was 45.9% (n = 1,536), while 54.1% (n = 1,813) showed no viral growth. Temporal series analysis revealed marked variations in both the absolute number of confirmed cases and the diagnostic positivity rate throughout the study period (Fig 1a). During the first half of 2023, a sustained increase in positive cases was observed, reaching a maximum peak in May 2023, with more than 500 confirmed cases in a single month. Subsequently, the absolute number of cases progressively declined during the second half of the same year. Despite this reduction in total case volume, the positivity rate remained high during specific intervals, exceeding 60% toward the end of 2023 and the beginning of 2024. Throughout 2024 and into 2025, the temporal trend exhibited a phase of relative stability, characterized by seasonal peaks of lower magnitude. During this period, the monthly number of positive cases ranged mainly between 100 and 150, with a punctual increase observed in February 2025 (Fig 1a**).**

#### 2.2.2. Temporal distribution and replacement dynamics of dengue virus serotypes.

Among the samples serotyped during the 2023–2025 period (n = 3,349), DENV-2 was the predominant serotype overall, accounting for 26.5% of samples (n = 889), followed by DENV-3 (10.9%; n = 364), DENV-4 (5.8%; n = 195), and DENV-1 (2.5%; n = 84). Mixed infections were uncommon (0.12%) and included combinations of DENV-1/DENV-2, DENV-2/DENV-3, DENV-2/DENV-4, and DENV-3/DENV-4 (Table 2).

**Table 2:** Prevalence of dengue virus positivity in clinical samples during the 2023–2025 outbreak in the Department of Meta (n = 3349).

	2023- 2025 (3349)	2023 (1782) (%)	2024 733(%)	2025 834(%)
DENV-1	84(2.5)	68(3.8)	16(2.18)	–
DENV-2	889(26.5)	686(38.49)	127(17.32)	76(9.11)
DENV-3	364(10.9)	111(6.22)	94(12.82)	159(19.06)
DENV-4	195(5.8)	15(0.84)	77(10.50)	103(12.35)
DENV-1 /DENV-2	1(0.02)	1(0.05)	–	–
DENV-2 /DENV-3	1(0.02)	1(0.05)	–	–
DENV-2 /DENV-4	1(0.02)	1(0.05)	–	–
DENV-3 /DENV-4	1(0.05)	1(0.05)	–	–
Negative	1813(54.1)	898(50.39)	419(57.16)	496(59.47)
Total	3349 (100)	1782 (100)	733(100)	834(100)

Absolute counts and percentages by dengue virus serotype for the entire study period and stratified by year (2023–2025). Individual serotype infections, mixed infections, and negative results are presented. DENV-1, DENV-2, DENV-3, and DENV-4 correspond to dengue virus serotypes 1, 2, 3, and 4, respectively. Combined categories represent mixed infections in which two serotypes were simultaneously detected in the same sample.

Marked changes in serotype circulation were observed throughout the study period (Fig 1b). During the epidemic peak in 2023, DENV-2 exceeded 60% of monthly viral isolates and clearly dominated serotype circulation. Beginning in mid-2024, DENV-3 progressively increased, accompanied by a decline in DENV-2 and a simultaneous expansion of DENV-4. By 2025, DENV-3 emerged as the leading circulating serotype and DENV-4 became the second most frequent serotype, whereas DENV-2 declined to 9.1% and DENV-1 was no longer detected. Notably, DENV-4 reached 103 isolates and represented 30.5% of all dengue-positive isolates identified in 2025.

Despite the documented circulation of the Cosmopolitan genotype of DENV-2 in the region, this serotype progressively declined and was displaced by DENV-3 and DENV-4, suggesting an active serotype replacement process in the department of Meta.

### 2.3. Spatial distribution and spatiotemporal changes in dengue positivity and serotype patterns

#### 2.3.1. Municipal distribution of dengue diagnostic positivity.

**Fig 2:**
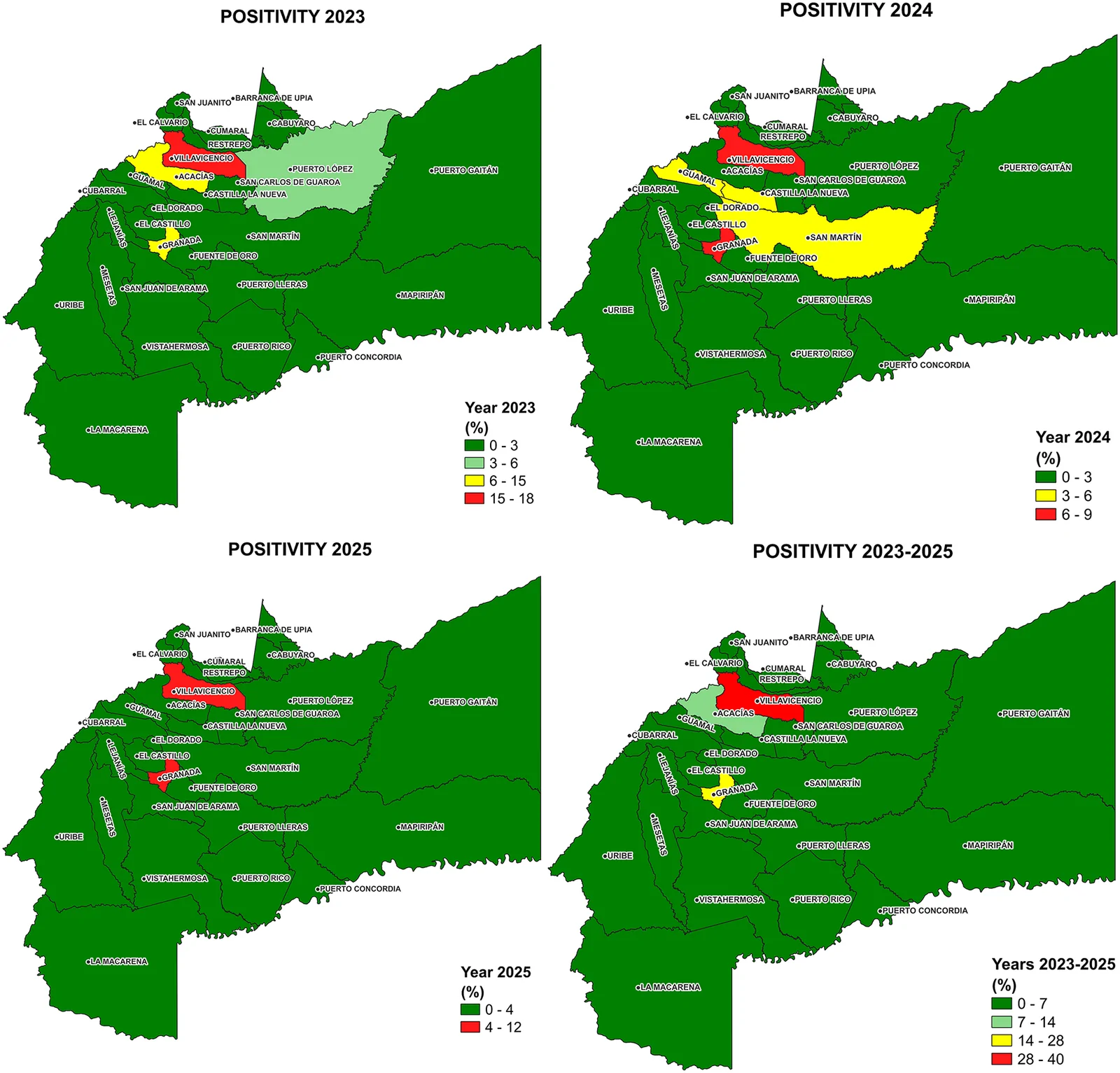
Spatial and temporal distribution of dengue diagnostic positivity at the municipal level in the Department of Meta, Colombia, during 2023–2025. Maps show the percentage ranges of diagnostic positivity by municipality for 2023, 2024, 2025, and for the cumulative study period (2023–2025). Municipal boundaries were derived from the official geo-statistical cartography of the National Administrative Department of Statistics (DANE), Colombia (https://geoportal.dane.gov.co/servicios/descarga-y-metadatos/datos-geoestadisticos/?cod=111). Data are distributed under the DANE Geoportal terms of use compatible with the Creative Commons Attribution 4.0 International License (CC BY 4.0) (https://geoportal.dane.gov.co/acerca-del-geoportal/licencia-y-condiciones-de-uso/). Maps were prepared using QGIS.

Fig 2 depicts the municipal distribution and temporal patterns of dengue diagnostic positivity in the Department of Meta during the 2023–2025 period. In 2023, the highest positivity values were recorded, with Villavicencio reaching approximately 15–18%, followed by Acacías and Granada. In contrast, several peripheral municipalities—including Mapiripán, Uribe, La Macarena, El Calvario, and San Juanito—remained within the lowest positivity ranges (0–3%). In 2024, maximum positivity values decreased across the department, with most municipalities falling within ranges of 0–3%. The highest values for that year were observed in Villavicencio and Granada, while Castilla La Nueva, Guamal and San Martín showed intermediate values (3–6%). In 2025, positivity values increased in Villavicencio and in Granada (8–12%), whereas most municipalities remained within low positivity ranges (0–4%). The cumulative analysis for the 2023–2025 period showed the highest aggregated positivity in Villavicencio (28–40%), followed by Granada (14–28%). Acacías presented intermediate cumulative values (7–14%), while most peripheral municipalities remained within the lowest cumulative ranges (0–7%).

### 2.4. Interannual changes in municipal dengue positivity

**Fig 3:**
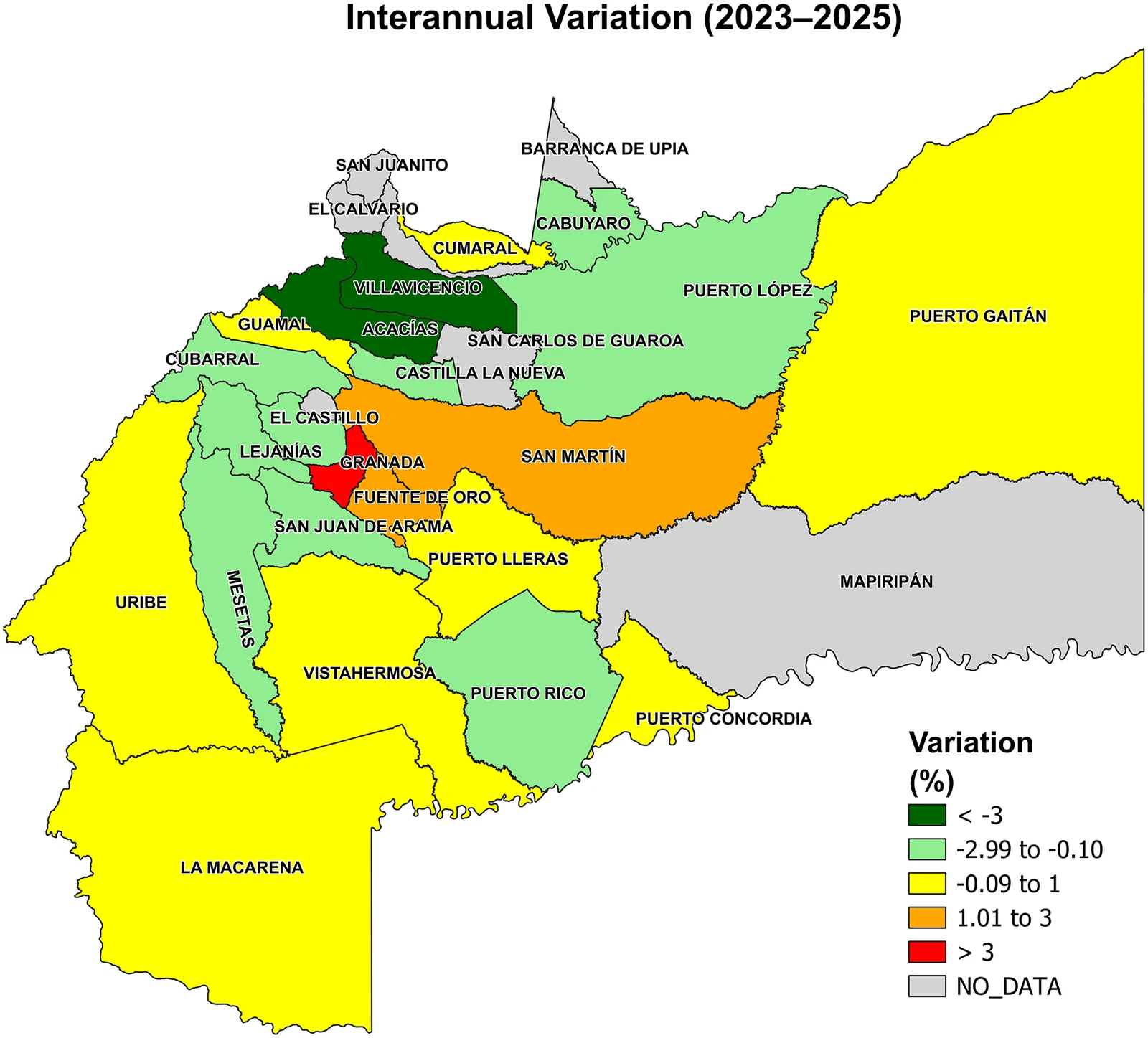
Interannual variation in dengue diagnostic positivity between 2023 and 2025 at the municipal level in the Department of Meta, Colombia. The map depicts the absolute change in positivity percentage points using a graduated scale, highlighting areas with decreases, stability, or increases in transmission during the study period. Municipal boundaries were derived from the official geo-statistical cartography of the National Administrative Department of Statistics (DANE), Colombia (https://geoportal.dane.gov.co/servicios/descarga-y-metadatos/datos-geoestadisticos/?cod=111). Data are distributed under the DANE Geoportal terms of use compatible with the Creative Commons Attribution 4.0 International License (CC BY 4.0) (https://geoportal.dane.gov.co/acerca-del-geoportal/licencia-y-condiciones-de-uso/). Maps were prepared using QGIS.

Overall, most municipalities with comparable data for 2023 and 2025 exhibited a reduction in transmission intensity, particularly those that had reported high positivity levels at the beginning of the study period (Fig 3). The most pronounced decreases were observed in Acacías (−6.77%), where positivity declined from 9.4% in 2023 to 2.6% in 2025, and Villavicencio (−5.12%), which decreased from 17.6% to 12.5%. Puerto López (−2.48%) also showed a notable decline, followed by Cubarral (−1.91%) and Lejanías (−1.09%), which were categorized as having a slight decrease in transmission. A second group of municipalities exhibited minor reductions or stable transmission, with interannual changes close to zero. This group included Castilla La Nueva, Puerto Rico, El Castillo, Cabuyaro, Mesetas, San Juan de Arama, and Uribe, with variations ranging from −0.70% to −0.07%, reflecting persistently low and relatively stable transmission levels throughout the study period. Similarly, La Macarena, Puerto Concordia, and Puerto Gaitán showed no interannual variation, maintaining constant positivity levels between both years.

In contrast, several municipalities demonstrated emerging and critical increases in transmission. Guamal (0.81%), Vista Hermosa (0.59%), and Cumaral (0.10%) showed slight increases, whereas Fuente de Oro (2.11%) and San Martín (2.86%) exhibited more pronounced increases classified as emerging transmission increases. The most substantial rise was identified in Granada (4.47%), where positivity increased from 7.1% in 2023 to 11.6% in 2025 and was therefore classified as a critical increase in transmission, suggesting a localized intensification of epidemiological risk.

Finally, the geospatial analysis identified a critical information gap in six municipalities (Mapiripán, Restrepo, El Dorado, among others) that lacked laboratory reports during 2025. These “silent zones” do not necessarily indicate an absence of transmission but rather an interruption in the flow of virological surveillance in peripheral areas. This phenomenon coincides with the emergence phase of the DENV-4 serotype, suggesting that the spread of this new serotype may be occurring in an underrecognized manner within low-endemicity nodes.

### 2.5. Spatial distribution patterns and co-circulation of DENV-1, DENV-2, DENV-3, and DENV-4 in the Department of Meta, Colombia (2023–2025)

**Fig 4:**
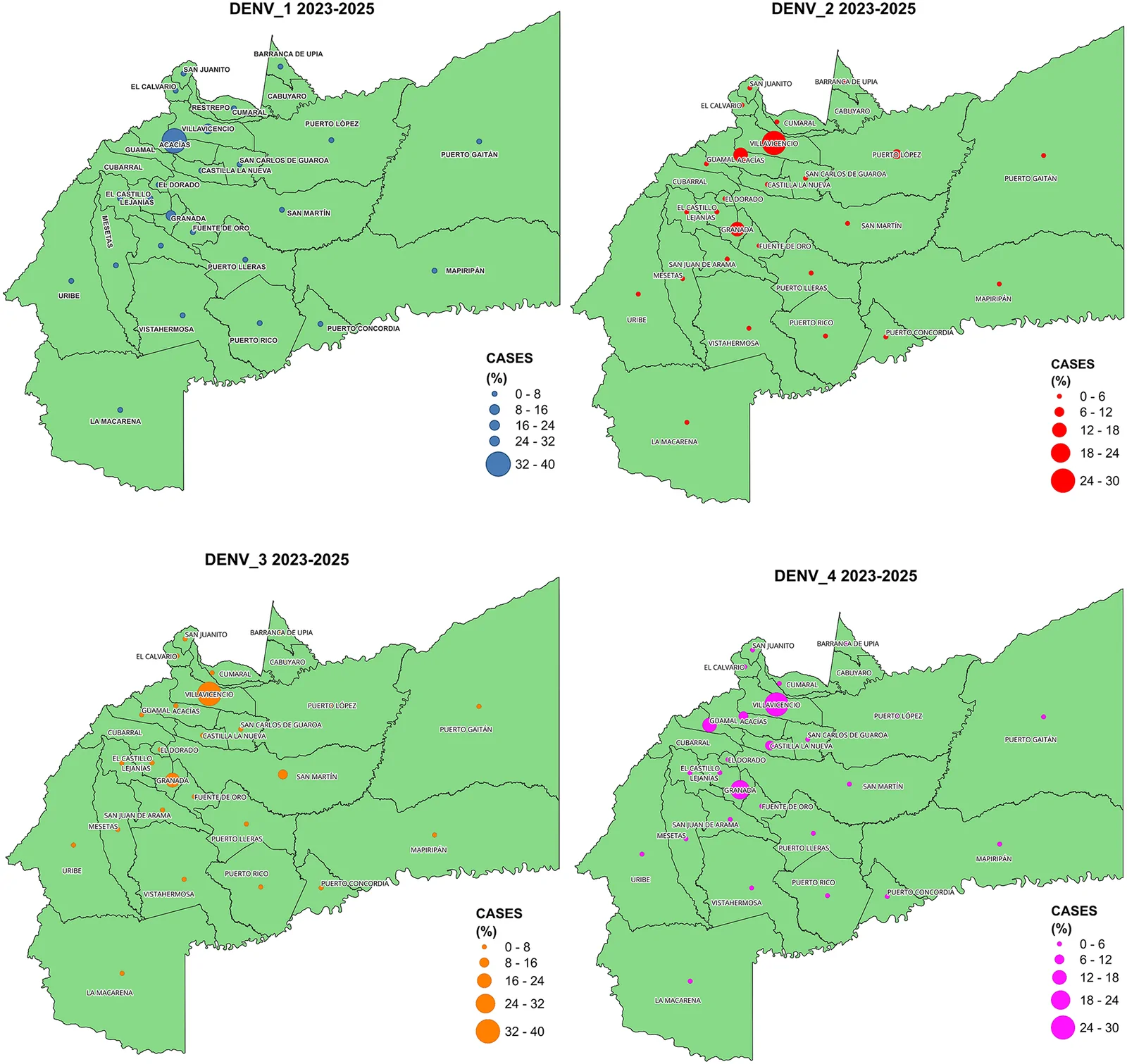
Spatial distribution patterns and co-circulation of DENV-1, DENV-2, DENV-3, and DENV-4 serotypes in the Department of Meta, Colombia (2023–2025). The maps depict the proportional incidence of each dengue virus serotype by municipality during the study period. Symbol size represents the percentage contribution of each serotype relative to the total number of typed samples. Municipal boundaries were derived from the official geo-statistical cartography of the National Administrative Department of Statistics (DANE), Colombia (https://geoportal.dane.gov.co/servicios/descarga-y-metadatos/datos-geoestadisticos/?cod=111). Data are distributed under the DANE Geoportal terms of use compatible with the Creative Commons Attribution 4.0 International License (CC BY 4.0) (https://geoportal.dane.gov.co/acerca-del-geoportal/licencia-y-condiciones-de-uso/). Maps were prepared using QGIS.

The cartographic assessment of dengue virus serotypes reveals distinct patterns of spatial distribution in the department of Meta, with Villavicencio and Granada identified as the main hotspots for all serotypes analyzed (Fig 4). Among the serotypes, DENV-2 stands out for its highest disease burden and territorial spread, reaching a maximum of 154–266 cases in the central cluster of Villavicencio and extending to peripheral municipalities such as Puerto López and Puerto Gaitán. DENV-3 shows an intermediate magnitude, peaking at up to 150 cases, primarily concentrated along the north-south axis of the department. DENV-4 with a range of 40–51 cases, predominantly in Villavicencio, Acacías, and Granada, whereas DENV-1 has the lowest case density, with a maximum of 24–30 cases in the main urban centers.

The dynamics of infection foci reveal three critical areas: the Northern Node (Metropolitan Area), including Villavicencio, Acacías, and Cumaral, which concentrates the highest population densities and the highest frequency of all serotypes; the Central Node (Ariari Region), led by Granada, maintaining consistent infection levels, particularly for DENV-2 and DENV-4; and the Low Endemicity Zones, comprising southern and eastern municipalities such as La Macarena, Uribe, and Mapiripán, where the lowest infection ranges (0–10 cases) are consistently observed across all serotypes.

## 3. Factors associated with dengue positivity and serotype distribution

### 3.1. Bivariate analysis of clinical and sociodemographic factors associated with dengue virus positivity and serotypes in the Department of Meta

#### 3.1.1. Determinants of dengue virus positivity: Bivariate epidemiological and clinical analysis.

The bivariate analysis demonstrated significant associations between viral positivity and temporal, sociodemographic, clinical, and geographic factors. Statistically significant differences were observed according to the year of notification (p < 0.001), with a higher proportion of positive cases in 2023 and a progressive decrease in 2024 and 2025. Male sex showed a lower probability of viral positivity compared with females (OR = 0.86; 95% CI: 0.75–0.99), as did individuals affiliated with the contributory health insurance scheme compared with those in the subsidized scheme (OR = 1.15; 95% CI: 1.00–1.31). Clinically, viral positivity was significantly associated with fever, headache, retro-orbital pain, myalgia, arthralgia, skin rash, abdominal pain, and emesis, as well as with increased hematocrit and thrombocytopenia <100,000 platelets/mm³, the latter showing the strongest association (OR = 2.74; 95% CI: 2.36–3.18; p < 0.001). General hospitalization was also associated with higher viral positivity (OR = 1.72; 95% CI: 1.45–2.03). In contrast, somnolence/irritability showed an inverse association with the outcome. Geographically, municipalities with lower odds of positivity (Acacías, Castilla La Nueva, Guamal, and Lejanías) and others with higher risk, particularly Cubarral, Granada, Mesetas, and Puerto Rico, were identified, suggesting relevant spatial heterogeneity in viral circulation (see S1 Table).

#### 3.1.2. Serotype-specific associations with clinical and epidemiological characteristics.

A bivariate analysis was conducted to evaluate the relationship between clinical and sociodemographic characteristics, considering the identified serotype as dependent variable, and clinical management, sociodemographic factors, and reported clinical signs and symptoms as independent variables (S2 Table). DENV-2 showed the strongest associations with clinical signs and laboratory parameters indicative of greater severity, such as thrombocytopenia, hemoconcentration, and hospitalization. In contrast, DENV-3 and DENV-4 demonstrated a progressive increase in frequency toward 2025, associated with moderate clinical presentations and greater symptomatic diversity.

- *DENV-1:* The presence of myalgia (OR = 3.07; p = 0.006; 95% CI: 1.33–7.08), as was severe mucosal bleeding (OR = 2.87; p = 0.012).
- *DENV-2:* associated with several clinical and laboratory manifestations. Associations were observed with headache (OR = 1.46; p < 0.001), myalgia (OR = 1.40; p = 0.001), arthralgia (OR = 1.50; p < 0.001), abdominal pain (OR = 1.26; p = 0.006), and emesis (OR = 1.47; p < 0.001). Additionally, significant associations were found with elevated hematocrit (OR = 1.81; p = 0.002), thrombocytopenia (OR = 2.44; p < 0.00), and hospitalization (OR = 1.67; p < 0.001).
- *DENV-3:* type of health insurance, being more frequent among patients in the subsidized system (OR = 1.30; p = 0.017;). Retroocular pain (OR = 1.36; p = 0.005), rash (OR = 1.50; p = 0.002), and thrombocytopenia (OR = 1.75; p < 0.001). Although infrequent, hypothermia showed a strong association with this serotype (OR = 5.15; p = 0.001). Likewise, hospitalization was more common among DENV-3 cases (OR = 1.33; p = 0.039).
- *DENV-4:* type of health insurance, being more frequent among patients in the subsidized system (OR = 1.43; p = 0.018) and myalgia showed a significant association (OR = 1.60; p = 0.029).

### 3.2. Multivariable binary logistic regression analysis of dengue virus infection

This section presents the results of the multivariable logistic regression models used to identify factors independently associated with dengue virus (DENV) positivity, as detailed in Table 3a, as well as factors associated with serotype distribution, shown in Table 3b, after adjustment for variables that were significant in the bivariate analysis.

**Table 3:** a. Logistic regression analysis for factors associated with dengue virus positivity.b. Logistic regression analysis for factors associated with dengue virus serotype.

a. Logistic regression analysis for factors associated with dengue virus positivity							
Predictor	B	SE	Z	p	Odds Ratio	95% CI	
						Lower	Upper
Year	-0.279	0.047	34.871	**<0.001**	0.756	0.689	0.830
Sex (1)	0.099	0.075	1.719	0.190	1.104	0.952	1.279
Health insurance type (1)	-0.045	0.076	0.360	0.548	0.956	0.824	1.108
Fever (1)	0.117	0.177	0.437	0.509	1.124	0.794	1.591
Headache (1)	-0.241	0.109	4.906	**0.027**	0.786	0.635	0.973
Retro-orbital pain (1)	-0.101	0,085	1.407	0.235	0.904	0.766	1.068
Myalgia (1)	-0.561	0.157	12.750	**<0.001**	0.571	0.420	0.777
Arthralgia (1)	0.203	0.168	1.458	0.227	1.225	0.881	1.702
Skin rash (1)	-0.319	0.099	10.385	**0.001**	0.727	0.599	0.882
Abdominal pain (1)	-0.085	0.089	0.898	0.343	0.919	0.771	1.095
Emesis (1)	-0.092	0.098	0.883	0.347	0.912	0.752	1.106
Somnolence /Irritability (1)	0.875	0.252	12.094	**0.001**	2.400	1.465	3.930
Hematocrit increase (1)	-0.157	0.201	0.608	0.436	0.855	0.577	1.268
Platelet count <100,000 (1)	-0.829	0.086	93.711	**<0.001**	0.437	0.369	0.516
hospitalization (1)	-0.138	0.104	1.742	0.187	0.871	0.710	1.069
Acacias (1)	0.172	0.123	1.935	0.164	1.187	0.932	1.513
Castilla la Nueva (1)	0.318	0.161	3.924	**0.048**	1.375	1.003	1.884
Cubarral (1)	-1.051	0.368	8.142	**0.004**	0.350	0.170	0.720
Granada (1)	-0.644	0.122	27.907	**<0.001**	0.525	0.414	0.667
Guamal (1)	0.093	0.184	0.256	0.613	1.097	0.765	1.573
Uribe (1)	-21.371	17623.748	0.000	0.999	0.000	0.000	
Lejanías (1)	0.788	0.248	10.121	**0.001**	2.200	1.353	3.575
Mesetas (1)	-1.844	0.783	5.548	**0.018**	0.158	0.034	0.734
Puerto Rico (1)	-0.926	0.426	4.738	**0.030**	0.396	0.172	0.912
Villavicencio (1)	-0.342	0.103	10.979	**0.001**	0.710	0.580	0.869
Constant	590.513	17624.008	0.001	0.973	3.E+256		
**b. Logistic regression analysis for factors associated with dengue virus serotype**							
DENV-1							
Year	-1.208	0.234	26.555	**<0.001**	0.299	0.189	0.473
Severe mucosal bleeding (1)	-1.065	0.461	5.326	**0.021**	0.345	0.140	0.852
Myalgia (1)	-1.355	0.430	9.920	**0.002**	0.258	0.111	0.599
Acacias (1)	-1.084	0.270	16.178	**<0.001**	0.338	0.199	0.574
Granada (1)	-1.372	0.299	21.087	**<0.001**	0.254	0.141	0.456
Mesetas (1)	-2.947	0.720	16.744	**<0.001**	0.052	0.013	0.215
Puerto López1)	1.603	1.021	2.466	0.116	4.969	0.672	36.756
San Martín (1)	0.599	1.029	0.339	0.560	1.821	0.242	13.676
Constant	2444.901	474.517	26.547	<0.001			
DENV-2							
Year	-0.877	0.063	193.045	**<0.001**	0.416	0.368	0.471
Headache (1)	-0.395	0.122	10.468	**0,001**	0.674	0.531	0.856
Myalgia (1)	-0.135	0.174	0.602	0.438	0.874	0.621	1.229
Arthralgia (1)	-0.045	0.193	0.054	0.816	0.956	0.655	1.396
Abdominal pain (1)	0.053	0.101	0.276	0..599	1.054	0.866	1.284
Emesis (1)	-0.115	0.108	1.127	0.288	0.892	0.722	1.102
Hematocrit increase (1)	-0.370	0.211	3.058	0.080	0.691	0.456	1.046
Platelet count <100,000 (1)	-0.827	0.096	74.798	**<0.001**	0.438	0.363	0.528
hospitalization (1)	-0,182	0.122	2.221	0.136	0.833	0.655	1.059
Castilla la Nueva (1)	0.557	0.185	9.044	**0.003**	1.745	1.214	2.509
Cubarral (1)	-1.019	0.336	9.223	**0.002**	0.361	0.187	0.697
Fuente de Oro(1)	0.917	0.368	6.199	**0.013**	2.502	1.216	5.151
Guamal (1)	0.619	0.261	5.622	**0.018**	1.856	1.113	3.096
Puerto Gaitán (1)	-0.521	0.212	6.033	**0.014**	0.594	0.392	0.900
Puerto López (1)	-0.166	0.155	1.135	0.287	0.847	0.625	1.149
Puerto Rico (1)	-1.299	0.419	9.614	**0.002**	0.273	0.120	0.620
San Carlos de Guaroa (1)	-0.991	0.679	2.129	0.145	0.371	0.098	1.405
San Martín (1)	0.758	0.250	9.218	**0.002**	2.135	1.308	3.483
Constant	1775.859	127.791	193.116	<0.001			
DENV-3							
Year	0.461	0.072	41.411	**<0.001**	1.585	1.378	1.824
Retro-orbital pain (1)	-0.143	0.120	1.422	0.233	0.867	0.685	1.096
Health insurance type (1)	-0.121	0.121	1.014	0.314	0.886	0.699	1.122
Skin rash (1)	-0.183	0.144	1.630	0.202	0.832	0.628	1.103
Hypothermia (1)	-1.639	0.593	7.632	**0.006**	0.194	0.061	0.621
Platelet count <100,000 (1)	-0.579	0.133	18.817	**<0.001**	0.561	0.432	0.728
Hospitalized (1)	0.098	0.164	0.362	0.548	1.103	0.801	1.520
Acacías (1)	1.072	0.346	9.590	**0,002**	2.920	1.482	5.753
Cubarral (1)	18.637	5882.614	0.000	0.997	1.E+08	0.000	
Fuente de Oro (1)	-1.339	0.300	19.859	**<0.001**	0.262	0.145	0.472
Granada (1)	-0.455	0.224	4.108	**0.043**	0.634	0.409	0.985
Guamal (1)	1.756	0.613	8.208	**0.004**	5.786	1.741	19.230
Uribe (1)	-2.033	0.955	4.526	**0.033**	0.131	0.020	0.852
Lejanías (1)	1.784	1.025	3.027	0,082	5.954	0.798	44.421
Puerto Gaitán1)	1.567	0.738	4.503	**0.034**	4.791	1.127	20.369
Puerto López (1)	0.270	0.361	0.559	0.455	1.310	0.646	2.658
San Martín (1)	-1.335	0.240	30.836	**<0.001**	0.263	0.164	0.422
Villavicencio (1)	-0.713	0.201	12.528	**<0.001**	0.490	0.330	0.728
Constant	-951.761	5884.396	0.026	0.872	0.000		
DENV 4							
Year	1.011	0.106	90.796	**<0.001**	2.748	2.232	3.383
Health insurance type (1)	-0.336	0.165	4.149	**0.042**	0.715	0.517	0.987
Myalgia (1)	-0.378	0.229	2.727	0.099	0.685	0.437	1.073
Cabuyaro (1)	-3.734	0.731	26.099	**<0.001**	0.024	0.006	0.100
El Dorado (1)	-1.805	0.891	4.109	**0.043**	0.164	0.029	0.942
Acacías (1)	0.052	0.305	0.029	0.865	1.053	0.579	1.915
Granada (1)	-0.560	0.198	8.029	**0.005**	0.571	0.387	0.841
Guamal (1)	-1.394	0.230	36.636	**<0.001**	0.248	0.158	0.390
Lejanías (1)	17.389	3810.960	0.000	0.996	35650203.348	0.000	
Mesetas (1)	-2.261	0.642	12.417	**<0.001**	0.104	0.030	0.367
Puerto Gaitán (1)	18.009	362.937	0.000	0.996	66283983.476	0.000	
Puerto López1)	1.168	0.726	2.592	0.107	3.216	0.776	13.335
Vistahermosa (1)	-1.203	0.409	8.637	**0.003**	0.300	0.135	0.670
Constant	-2074.3	5261.922	0.155	0.693	0.000		

Table 3a. Adjusted logistic regression analysis of factors associated with dengue virus positivity in the Department of Meta, Colombia (2023–September 2025). Results are expressed as regression coefficients (B), standard errors (SE), Wald Z statistics (Z), odds ratios (ORs), and 95% confidence intervals (95% CI). Statistical significance was defined as p < 0.05.

Table 3b. Adjusted logistic regression models for factors associated with dengue virus serotypes (DENV-1–DENV-4) in the Department of Meta, Colombia (2023–September 2025). Results are expressed as regression coefficients (B), standard errors (SE), Wald Z statistics (Z), odds ratios (ORs), and 95% confidence intervals (95% CI).

Footnote: High accuracy versus low sensitivity reflects the early emergence phase of DENV-4 and its low prevalence during the initial period of observation.

### 3.3. Predictors of Dengue Virus (DENV) Positivity

Based on the variables that were significant in the bivariate analysis for dengue positivity (S1 and S2 Table), the multivariate analysis showed that the year of notification was independently associated with dengue positivity (OR = 0.75; p < 0.001). Among clinical variables, several classical manifestations of dengue, including headache (OR = 0.78; p = 0.027), myalgia (OR = 0.57; p < 0.001), and skin rash (OR = 0.72; p = 0.001), were inversely associated with virological confirmation. In contrast, nonspecific neurological manifestations, particularly somnolence and irritability, emerged as the strongest independent clinical predictor of laboratory-confirmed dengue (OR = 2.41; p = 0.001). This finding suggests that emerging clinical phenotypes may provide greater discriminatory value than several traditional manifestations in the current epidemiological context. A platelet count <100,000/µL showed a significant association with dengue positivity (OR = 0.43; p < 0.001). At the geographic level, higher probabilities of positivity were observed in Castilla la Nueva and Lejanías, and lower probabilities in Cubarral, Granada, Mesetas, Puerto Rico, and Villavicencio. The model was globally significant (χ² = 380.48; df = 25; p < 0.001), with moderate explanatory power (Cox and Snell R² = 0.107; Nagelkerke R² = 0.144) and an overall classification accuracy of 65.2%, with better performance in identifying negative cases (76.2%) than positive cases (52.2%)

#### 3.3.1. Serotype-specific multivariable logistic regression analysis.

- *DENV-1*: In the adjusted analysis, the year of notification was associated with a lower probability of DENV-1 infection (OR = 0.30; p < 0.001). The presence of severe mucosal bleeding (OR = 0.35; p = 0.021) and myalgia (OR = 0.26; p = 0.002) were inversely associated with DENV-1 confirmation. From a geographic perspective, residence in the municipalities of Acacías (OR = 0.34; p < 0.001), Granada (OR = 0.25; p < 0.001), and Mesetas (OR = 0.05; p < 0.001) was associated with a lower probability of DENV-1 infection. The multivariate logistic regression model for the DENV-1 serotype was globally significant according to the omnibus tests of model coefficients (χ² = 106.48; df = 8; p < 0.001). The remaining variables included in the model did not reach statistical significance after multivariate adjustment. Model fit indicated low to moderate explanatory power, with a Cox and Snell R² of 0.031 and a Nagelkerke R² of 0.149. The overall predictive accuracy reached 97.5%.
- *DENV2:* In the adjusted analysis, the year of notification (OR = 0.42; p < 0.001), headache (OR = 0.67; p = 0.001), and a platelet count <100,000/µL (OR = 0.44; p < 0.001) were significantly associated with DENV-2 infection. Higher probabilities of infection were observed in Castilla la Nueva (OR = 1.75; p = 0.003), Fuente de Oro (OR = 2.50; p = 0.013), Guamal (OR = 1.86; p = 0.018), and San Martín (OR = 2.14; p = 0.002). In contrast, lower probabilities of infection were identified in Cubarral (OR = 0.36; p = 0.002), Puerto Gaitán (OR = 0.59; p = 0.014), and Puerto Rico (OR = 0.27; p = 0.002). The remaining variables included in the model did not reach statistical significance after multivariate adjustment. The model was globally significant according to the omnibus tests of model coefficients (χ² = 534.69; df = 18; p < 0.001) and showed moderate explanatory power, with a Cox and Snell R² of 0.148 and a Nagelkerke R² of 0.215.
- *DENV3:* The year of notification was significantly associated with a higher probability of DENV-3 infection (OR = 1.59; p < 0.001), suggesting a temporal increase in the circulation of this serotype. In contrast, hypothermia (OR = 0.19; p = 0.006) and a platelet count <100,000/µL (OR = 0.56; p < 0.001) were inversely associated with DENV-3 positivity. Higher odds of DENV-3 infection were observed in the municipalities of Acacías (OR = 2.92; p = 0.002), Guamal (OR = 5.79; p = 0.004), and Puerto Gaitán (OR = 4.79; p = 0.034). Conversely, inverse associations were identified in Fuente de Oro (OR = 0.26; p < 0.001), Granada (OR = 0.63; p = 0.043), Uribe (OR = 0.13; p = 0.033), San Martín (OR = 0.26; p < 0.001), and Villavicencio (OR = 0.49; p < 0.001). The remaining variables included in the model did not reach statistical significance after adjustment. The model was globally significant according to the omnibus tests of model coefficients (χ² = 308.59; df = 18; p < 0.001). Model fit indicated a low to moderate explanatory capacity, with a Cox and Snell R² of 0.088 and a Nagelkerke R² of 0.177. Overall predictive accuracy was 89.0%, with a high correct classification of negative cases (99.8%) and low sensitivity for positive cases (0.8%).
- *DENV4:* In the adjusted analysis, the year of notification was significantly associated with a higher probability of DENV-4 infection (OR = 2.75; p < 0.001). Health insurance type was inversely associated with DENV-4 positivity (OR = 0.72; p = 0.042). Significant inverse associations were observed in Cabuyaro (OR = 0.02; p < 0.001), El Dorado (OR = 0.16; p = 0.043), Granada (OR = 0.57; p = 0.005), Guamal (OR = 0.25; p < 0.001), Mesetas (OR = 0.10; p < 0.001), and Vistahermosa (OR = 0.30; p = 0.003). The remaining variables included in the model did not reach statistical significance after multivariable adjustment. The logistic regression model was globally significant according to the omnibus tests of model coefficients (χ² = 250.42; df = 13; p < 0.001). Model fit showed a low to moderate explanatory capacity, with a Cox and Snell R² of 0.072 and a Nagelkerke R² of 0.200. Overall classification accuracy was 94.1%, with excellent discrimination of negative cases (99.9%) but very low sensitivity for positive cases (1.0%), indicating limited predictive performance for identifying DENV-4 positive cases despite the high overall accuracy.

This statistical imbalance is expected in the context of an emerging pathogen, where the low prevalence of positive cases during the initial phase of serotype replacement (early 2025) limits the model’s ability to correctly classify the minority class. Nevertheless, the high specificity and the statistical significance of the identified odds ratios (OR: 2.75) confirm the robustness of the predictors despite the incipient stage of the outbreak.

## 4. Spatiotemporal dynamics and multivariable risk integration: The DENV-4 Emergence and Geographic Rebound

The integration (Table 4) of adjusted logistic regression models (Table 3) and Spatiotemporal Analysis and Interannual Variation (Fig 3) reveals a significant shift in dengue dynamics in the Department of Meta between 2023 and September 2025.

**Table 4:** Integration of Geographic Persistence and Statistical Risk Predictors (2023–2025).

Municipality	Interannual Var. (%)	Epidemiological Status	Adjusted OR (95% CI)	Key Serotype Association
Villavicencio	-5.12%	Marked decrease	0.71 (0.57-0.88) *	Negative association DENV-3) *
Acacias	-6.77%	Marked decrease	1.18 (0.84-1.68)	Positive association DENV-3) *
Granada	+4.47%	Critical increase	0.52 (0.35-0.78) *	-
San Martín	+2-86	Emerging increase	N/A	High Risk for DENV-2 (OR= 2.13) *
Fuente de Oro	+2.11	Emerging increase	N/A	High Risk for DENV-2 (OR= 2.50) *
Puerto Gaitán	0.00%	Stable	N/A	Association whit DENV-3 (OR=4.79)
Castilla La Nueva	-0.86		1,37	High Risk for DENV2 (OR= 1.74) *
Clinical predictors				
Top clinical predictors	Shift 2023-2025	Impact	Adjusted OR (95% CI)	Significance
Somnolence/irritability	New Dominant sign	High Severity	2.40(1.46-3.94)	P=0.001
Myalgia	Decreasing Trend	Clinical marker	0,57(0.42-0.77)	<0.001

*Significant at p < 0.05

This table synthesizes municipal level interannual variation in diagnostic positivity with adjusted odds ratios (ORs) derived from multivariable models, integrating epidemiological status and dominant serotype associations. Interannual variation (%) reflects changes between 2023 and 2025 and is categorized into marked decrease, emerging increase, critical increase, or stability. Adjusted ORs with 95% confidence intervals (CI) indicate the magnitude and direction of association with dengue risk; statistically significant estimates are denoted by an asterisk (*). Serotype associations summarize predominant or emerging DENV serotypes linked to increased or decreased risk at the municipal level. The lower panel presents key clinical predictors, highlighting temporal shifts in dominance (2023–2025), their clinical impact, corresponding adjusted ORs (95% CI), and statistical significance.

- Temporal Risk and Serotype Replacement: While the multivariable model indicates a general decline in the odds of positivity over time (OR: 0.756; p < 0.001), this trend is countered by the aggressive emergence of DENV-4. This serotype exhibited a 2.748-fold increase in odds per year (p < 0.001), fundamentally reconfiguring the regional risk map.
- Geospatial Remission in Urban Hubs: The interannual variation (Fig 3) shows a Marked decrease in transmission in historically hyper-endemic centers. Acacías recorded the most significant reduction in the department with -6.77% (dropping from 9.4% to 2.60%), followed by the capital, Villavicencio, which showed a -5.12% decrease. The multivariable analysis supports this remission, with Villavicencio showing a lower adjusted OR for positivity (0.710; 95% CI: 0.580–0.869; p = 0.001).
- The Rebound Effect in Secondary Nodes: In contrast to the capital, a critical “rebound” was identified in the Central Node (Ariari Region). Granada moved from a baseline positivity of 7.1% in 2023 to 11.60% in 2025, representing a critical increase of +4.47%. This expansion is geographically linked to neighboring municipalities like San Martín (+2.86%) and Fuente de Oro (+2.11%), where the latter remained a significant risk node in the multivariable models.
- Municipalities with negative interannual variation, such as Acacías, Villavicencio, and Cubarral, exhibited substantial reductions in diagnostic positivity, accompanied in most cases by serotype-specific odds ratios (ORs) below unity. This pattern suggests a protective effect or a reduction in the effective circulation of specific serotypes, particularly DENV-2 and DENV-3. In contrast, municipalities with interannual increases, such as Granada, Fuente de Oro, and San Martín, displayed heterogeneous serotype association patterns. Notably, Fuente de Oro and San Martín showed elevated ORs for DENV-2, indicating localized hotspots of increased virological risk.
- Clinical Predictors of the Shift: The emergence of these hotspots coincided with a distinct change in the clinical profile. Somnolence and irritability emerged as the strongest predictors for positivity (OR: 2.400; 95% CI: 1.465–3.930; p = 0.001). Conversely, classical symptoms like myalgia showed a decreasing trend (OR: 0.571; p < 0.001), suggesting a transition toward more severe clinical markers during the DENV-4 replacement phase.
- Surveillance Gaps: Geospatial analysis identified six municipalities, including Mapiripán, Restrepo, and El Dorado, with Insufficient data for analysis (No data) in 2025. These silent zones represent a critical challenge for the surveillance system, as the lack of laboratory reporting in peripheral areas may mask undetected viral circulation.

## 5. Discussion

This retrospective longitudinal observational study provides a comprehensive characterization of the dengue outbreak that occurred in the department of Meta between 2023 and September 2025, integrating clinical, epidemiological, virological, and spatial information derived from routine surveillance records. The findings demonstrate that outbreak dynamics were not determined exclusively by transmission magnitude, but rather by a complex interaction between serotype transition, territorial redistribution of risk, and transformation of the clinical profile associated with infection—factors widely recognized as critical determinants of dengue epidemiology in hyperendemic settings [17,20–22].

### 5.1. Temporal dynamics and serotype transition

One of the central findings of this study is the evidence of a progressive serotype replacement over the analyzed period. The marked predominance of DENV-2 during 2023, coinciding with the epidemic peak, was followed by a gradual decline in its circulation and a sustained increase in DENV-3 and, notably, DENV-4 toward 2025. The disappearance of DENV-1 in the most recent records reinforces the hypothesis of a structural virological reorganization rather than random fluctuations.

This pattern is consistent with previously described immuno-epidemiological models, in which population immunity acquired against a dominant serotype generates selective pressures that favor the expansion of alternative serotypes, increasing virological heterogeneity and the risk of secondary infections [13,23,24]. In Colombia, this phenomenon is particularly relevant in the context of the recent introduction of the cosmopolitan genotype of DENV-2, whose high dispersal capacity has been documented and may have contributed to modifying local transmission dynamics [5].

The relative emergence of DENV-4 observed in 2025 is especially relevant, given that this serotype has historically circulated at low levels in the country. Although serotype-specific models showed low predictive sensitivity, the significant temporal increase suggests an early phase of expansion that could precede larger outbreaks, particularly in populations with low prior immunity to this serotype [15, 22]. The discrepancy between the high accuracy and low sensitivity in the DENV-4 predictive model reflects a classic ‘class imbalance’ problem, typical of real-world epidemiological surveillance during the redistribution of a serotype. At this stage, the model is highly effective at identifying who does not have DENV-4 (high specificity), which is crucial for differential diagnosis in hyperendemic settings. As DENV-4 continues to displace DENV-2, we anticipate that the sensitivity of clinical predictors will increase, as the clinical phenotype becomes more consolidated within the population [14].

Our findings suggest a complex epidemiological transition: while the introduction of the Cosmopolitan genotype of DENV-2 may have driven the initial epidemic peak in 2023, its subsequent decline coincides with a shift in the clinical phenotype of the region. The emergence of DENV-4 in 2025 represents not only a serotype replacement but also a challenge to classical detection algorithms, as the greater symptomatic diversity observed may reflect an adaptive viral response to a population with pre-existing heterologous immunity to DENV-2 variants.

### 5.2. Persistence of transmission and active endemicity

Although multivariable analysis showed an overall reduction in the probability of positivity over time, the persistence of high diagnostic positivity rates even during periods with lower case volumes suggests a scenario of active endemicity rather than episodic transmission (Fig 1a). This behavior has been described in other Latin American countries, where alternating epidemic peaks and phases of sustained transmission reflect the continuous interaction among climatic factors, vector density, and population susceptibility [25–29].

In this context, the results support recommendations from the Pan American Health Organization and the Colombian National Institute of Health, which emphasize the need to maintain year-round active surveillance systems and avoid exclusively seasonal approaches that could delay early detection of changes in viral circulation[2,4].

### 5.3. Spatial redistribution of risk and epidemiological rebound

Spatial analysis revealed a significant redistribution of transmission risk at the municipal level. Historically hyperendemic municipalities such as Villavicencio and Acacías showed a marked reduction in diagnostic positivity, consistent with a phase of relative remission. In contrast, municipalities located in the central axis of the department—particularly Granada, San Martín, and Fuente de Oro—experienced significant interannual increases, configuring an “epidemiological rebound” pattern outside the main urban centers.

This territorial shift in risk has been previously described in scenarios where vector control strategies are disproportionately concentrated in large cities, favoring the persistence or emergence of secondary foci that are less visible but epidemiologically relevant [30–32]. These findings underscore the need for dynamic, territorially adaptive approaches capable of responding to changes in spatial transmission patterns.

### 5.4. Transformation of the dengue clinical profile and challenges for diagnostic confirmation

One of the most relevant findings of this study is the transformation of the clinical profile associated with virological confirmation of dengue. In multivariable models, classical symptoms historically considered highly predictive—such as headache, myalgia, and rash—showed inverse associations with viral positivity, suggesting a progressive loss of clinical specificity in hyperendemic settings. In contrast, nonspecific neurological manifestations, such as somnolence and irritability, emerged as the most robust independent clinical predictors of confirmed infection.

This shift in the clinical phenotype has been described in recent dengue and arbovirus outbreaks, particularly in scenarios characterized by the cocirculation of multiple serotypes and a high proportion of secondary infections, where heterologous immunological memory modulates the clinical response and alters the classical presentation of the disease [16,33]. In this context, traditional symptoms lose discriminatory value, while subtle systemic or neurological manifestations acquire greater epidemiological and diagnostic relevance.

From an operational perspective, these findings have direct implications for clinical surveillance. The continued reliance on diagnostic algorithms based exclusively on classical criteria may lead to underestimation of cases and delays in virological confirmation in dynamic epidemic scenarios. Therefore, it is necessary to review and update clinical suspicion algorithms, incorporating broader and more adaptive symptomatic profiles that are consistent with the evolving immuno-epidemiological landscape of dengue in hyperendemic regions.

### 5.5. Thrombocytopenia as a consistent marker of dengue severity

Despite the observed clinical variability, severe thrombocytopenia remained the biological marker most consistently associated with dengue infection, both in the overall analysis and in serotype-specific models, particularly for DENV-2. This finding is consistent with dengue pathophysiology, in which platelet dysfunction and endothelial damage play a central role regardless of the circulating serotype [34–36]. In a context of increasingly heterogeneous clinical presentations, the stability of this marker reinforces its value for objective risk stratification.

The most striking finding for public health surveillance is the early evidence of clinical ‘dilution’ of classic symptoms. As DENV-4 establishes dominance, the reliance on headache or myalgia for suspected cases may lead to significant underdiagnosis. We propose that somnolence and irritability should be prioritized in clinical algorithms, to ensure early detection of cases during this new virological wave, as well as in other high-risk groups, including older adults, pregnant women, and patients with underlying comorbidities.

### 5.6. Serotype-specific heterogeneity and differentiated surveillance

Serotype-specific models revealed differentiated epidemiological profiles. DENV-2 maintained consistent associations with hospitalization and markers of greater severity, whereas DENV-3 and DENV-4 showed more focal geographic patterns and more variable clinical associations. The low predictive sensitivity observed for emerging serotypes, despite high overall accuracy, is consistent with early phases of serotype expansion and supports the need to integrate virological and, when feasible, genomic information into surveillance systems [14,37].

### 5.7. Surveillance gaps and silent zones

The identification of “silent zones” in peripheral municipalities constitutes one of the most concerning findings from a regional health security perspective [2,38,39]. More than a technical limitation, this lack of data reveals a structural vulnerability in the surveillance system that favors “silent transmission.” In a context of serotype replacement—where DENV-4 shows an exponential increase in the odds of infection (OR: 2.75)—the blinding of the diagnostic network in areas such as Mapiripán or El Dorado prevents the timely detection of emerging outbreaks and the characterization of new clinical phenotypes outside urban centers. For public health authorities, this implies that vector control strategies based solely on data from hyperendemic centers may be overlooking critical viral reservoirs that sustain the persistence of the epidemic across the department. In scenarios of serotype cocirculation and emergence of new lineages, such gaps may facilitate undetected viral spread, as documented in other peripheral regions of Latin America [2,40]. In the case of Mapiripán, it can be inferred that it is a municipality with low population density, dispersed rural areas, and the presence of criminal groups—factors that interfere with access to the health system and the reporting of cases [41,42].

### 5.8. Strengths and limitations

This study is based on routine surveillance data generated by the Meta Public Health Laboratory and is supported by the processing of 3,349 samples, which constitutes one of its main strengths. The large sample size, the serotype identification, and the integration of multivariable and spatial analyses confer high external validity to the findings and allow for a robust assessment of dengue epidemiological dynamics in the region. Nevertheless, limitations inherent to this retrospective longitudinal observational study based on routinely collected surveillance data must be acknowledged. There is a potential for selection and reporting biases arising from the uneven distribution of samples across municipalities, especially in peripheral areas. The presence of “No Data” categories in certain territories likely reflects logistical challenges in sample transportation, underreporting, or variability in the quality of clinical records, rather than a true absence of viral circulation. In addition, incomplete information in some municipalities constrains fine-scale spatial interpretation and limits the ability to establish causal relationships. Although the study identifies a transition toward DENV-4, an important limitation is the lack of genomic characterization of the 2025 isolates. This precludes confirmation of whether the replacement of the Cosmopolitan DENV-2 is due to the introduction of a new DENV-4 genotype with greater biological fitness or to local genetic drift. A limitation of our statistical approach is the low sensitivity of the DENV-4 model. This is inherently linked to the period of study, which caught the very beginning of the DENV-4 upsurge. While this limits the model’s current use as a standalone screening tool, the statistical significance of the associations provides a valuable ‘early warning’ of the changing clinical landscape in the region.

Rather than undermining the results, these limitations highlight the need to strengthen decentralized laboratory networks and surveillance systems in the region, particularly during periods of serotype replacement, when timely and homogeneous detection is critical for informed public health decision-making.

## 6. Conclusion

Overall, this study demonstrates that the dengue outbreak in the department of Meta between 2023 and 2025 was characterized by a dynamic serotype transition, a spatial redistribution of transmission risk, and a transformation of the clinical profile associated with infection. The rapid emergence of DENV-4 in 2025 represents a critical shift in the local epidemiological landscape and acts as an early warning signal of serotype replacement. This transition, together with the epidemiological rebound observed in intermediate municipalities and the progressive loss of specificity of classical dengue symptoms, defines a changing scenario that challenges traditional surveillance and control approaches. In this context, our findings underscore the urgent need for integrated surveillance systems that are sensitive to virological, territorial, and clinical dynamics, including the incorporation of non-specific neurological markers into clinical screening protocols in hyperendemic regions. Finally, closing surveillance gaps in low-endemicity areas and strengthening decentralized laboratory networks emerge as strategic priorities, not only for Meta but also for other hyperendemic regions undergoing similar virological transitions; this includes reinforcing surveillance in epidemiologically silent municipalities such as Mapiripán and El Dorado.

## 7. Methods

Ethical considerations and permissions: The study was approved by the Bioethics Committee of the Universidad Cooperativa de Colombia (approval No. BIO785, April 23, 2025). Since the analysis was based on routine surveillance data identified by unique codes, data use authorization was granted by the Bioethics Committee, and individual informed consent was waived in accordance with current regulations. Nonetheless, strict confidentiality and data security measures were implemented, including encrypted data storage and restricted access to authorized research personnel.

Study design and population: A retrospective longitudinal observational study based on routinely collected surveillance data was conducted between January 1, 2023, and September 30, 2025, during the dengue outbreak period in the Department of Meta, Colombia. The study analyzed temporal, spatial, clinical, and virological patterns among all reported suspected dengue cases with available laboratory testing and serotype information.

**Reporting guideline:** This study was reported in accordance with the RECORD (Reporting of studies Conducted using Observational Routinely-collected health Data) statement, an extension of the STROBE guideline developed for studies using routinely collected health data. The completed RECORD checklist is provided as Supplementary Material.

Setting and data sources: Data were obtained from two main sources provided by the Meta Departmental Health Secretariat: the epidemiological notification form and the database of the Public Health Laboratory–Meta, used for arbovirus surveillance and containing diagnostic test results (RT-PCR Nucleic Acid Detection, NS1, or serology). Both data sources were linked using a unique case notification code. The investigators had access to an anonymized dataset containing routinely collected epidemiological and laboratory surveillance records from the Public Health Laboratory and the departmental surveillance system of Meta, Colombia. No direct patient identifiers were available. Before analysis, the databases underwent a structured data-cleaning process that included verification of record completeness, removal of duplicate entries, standardization of variable coding, and consistency checks between epidemiological and laboratory records. Records with incomplete laboratory information or without available serotype data were excluded from serotype-specific analyses according to predefined eligibility criteria.

Inclusion and exclusion criteria: Records were included if they met all the following criteria: (i) notification of a suspected dengue case in the epidemiological form; (ii) registration in the Public Health Laboratory database with at least one diagnostic result (RT-PCR, NS1 antigen detection, or serology); and (iii) availability of dengue virus serotyping results. Of the 3,717 suspected dengue cases analyzed during the study period, 3,349 met these criteria and had complete virological information, including dengue virus serotyping performed by RT-PCR-based nucleic acid detection. A total of 368 records were excluded from serotype-specific analyses because serotype information was unavailable, including 91 cases with only NS1 antigen results, 249 cases with only IgM ELISA results, and 28 records with incomplete laboratory information. These records could not be assigned to a specific dengue virus serotype and were therefore not eligible for analyses of serotype circulation, replacement dynamics, or spatial distribution (S1 Fig).

Laboratory diagnosis and serotype determination: Laboratory information was obtained exclusively from the surveillance database of the Public Health Laboratory of Meta Department. Diagnostic records included results from RT-PCR, NS1 antigen detection, and IgM serology, which were performed as part of routine dengue surveillance according to the standardized protocols established by the Colombian National Institute of Health (INS). Dengue virus serotype information (DENV-1, DENV-2, DENV-3, and DENV-4) was determined exclusively by RT-PCR and obtained from routine virological surveillance records. The present study did not involve the collection of biological samples or the performance of additional laboratory analyses, and all virological and serotype information was derived from existing surveillance records.

Variables collected:

- Sociodemographic: age, sex, municipality of residence, and type of health insurance coverage.
- Clinical: date of symptom onset, signs and symptoms, final clinical classification, diagnostic result (positive/negative), diagnostic technique (RT-PCR, NS1, IgM/IgG), dengue virus serotype (DENV-1 to DENV-4), clinical management, and outcomes (outpatient care, observation, hospitalization, or intensive care unit admission).

### 7.1. Data analysis

Statistical analyses: Statistical analyses were performed using SPSS software. Frequencies and percentages were calculated, and bivariate analyses were conducted to assess associations between dengue virus positivity, serotype distribution, and demographic or clinical variables. Statistical significance was determined using the chi-square test (p < 0.05), and odds ratios (ORs) with 95% confidence intervals were estimated. For binary variables, the first category listed in each contingency table was used as the reference category for odds ratio estimation; therefore, ORs represent the odds of the second category relative to the first category. To control for potential confounding factors, multivariable logistic regression models were fitted, including variables that showed statistical significance in the bivariate analysis.

Spatial Analysis and Georeferencing of Dengue Cases (2023–2025): The spatial distribution of dengue cases was analyzed using thematic maps in QGIS for scientific visualization and analytical purposes. Positivity maps depicted the proportion of positive samples per municipality and year, with three individual maps corresponding to 2023, 2024, and 2025 arranged in a comparative panel, along with an aggregated map for the 2023–2025 period. A sequential color scale from green (lowest positivity) to red (highest positivity) was applied. Serotype distribution (DENV-1 to DENV-4) was represented using circle sizes scaled proportionally to the detection percentage in each municipality. An interannual variation map was constructed to assess spatial changes in dengue transmission intensity at the municipal level. Interannual variation (VAR) was calculated as the difference between the percentage of positive diagnostic results in the final year and the initial year of the study period (VAR = POS2025 – POS2023). To avoid artificial distortion of persistence and trend patterns, municipalities lacking laboratory-confirmed data for the final year (2025) were excluded from the spatial analysis. This approach ensured that the spatial hotspots depicted in Fig 3 reflect only municipalities with continuous, laboratory-verified surveillance throughout the study period. A diverging color scale was applied to represent the interannual variation in transmission intensity: values below −3.00% were classified as a marked decrease in transmission (dark green); values between −2.99% and −0.10% as a slight decrease (light green); values between −0.09% and 1.00% as stable transmission (yellow); values between 1.01% and 3.00% as an emerging increase (orange); and values above 3.00% as a critical increase in transmission (red). Municipalities with insufficient data for analysis were displayed in light grey.

## Supporting information

S1 Checklist RECORD checklist completed for the present study. (DOCX)

S1 Fig Flow diagram of study selection and inclusion process. (DOCX)

S1 Table Bivariate analysis of clinical and sociodemographic factors associated with viral positivity. (DOCX)

S2 Table Bivariate analysis of clinical and sociodemographic factors associated with serotypes in the Department of Meta. S1 and S2 Tables. Bivariate analyses of demographic, clinical, and laboratory characteristics associated with dengue diagnostic positivity and dengue virus serotype distribution in the Department of Meta, Colombia, 2023–2025.
(DOCX)
