# Supplementary material for: Early warning of serotype replacement, spatial risk redistribution, and shifting clinical phenotypes: Insights from the 2023–2025 dengue outbreak in Meta, Colombia
Source: PLoS Negl Trop Dis. 2026 Sep 25;20(9):e0014733. doi: 10.1371/journal.pntd.0014733 (PMC13614597; doi:10.1371/journal.pntd.0014733)
Supplement: Supplementary file 1 [file pntd.0014733.s001.docx]

# The RECORD statement – checklist of items, extended from the STROBE statement, that should be reported in observational studies using routinely collected health data.

|  | **Item No.** | **STROBE items** | **Location in manuscript where items are reported** | **RECORD items** | **Location in manuscript where items are reported** |
| --- | --- | --- | --- | --- | --- |
| **Title and abstract** | | | | | |
|  | 1 | (a) Indicate the study’s design with a commonly used term in the title or the abstract (b) Provide in the abstract an informative and balanced summary of what was done and what was found | Title and Abstract, lines 1–49 | RECORD 1.1: The type of data used should be specified in the title or abstract. When possible, the name of the databases used should be included.  RECORD 1.2: If applicable, the geographic region and timeframe within which the study took place should be reported in the title or abstract.  RECORD 1.3: If linkage between databases was conducted for the study, this should be clearly stated in the title or abstract. | Abstract – Methods, lines 31–37.  Methods – Study Design and Data Sources, lines 625–628.  Title and Abstract, lines 25–35.  Introduction – Study Objectives, lines 133–141.  Methos, **Study design and population, lines 615- 618**  **Setting and data sources line 625** |
| **Introduction** | | | | | |
| Background rationale | 2 | Explain the scientific background and rationale for the  investigation being reported | Introduction, lines 69–145 |  |  |
| Objectives | 3 | State specific objectives, including any prespecified hypotheses | Introduction – Study Objectives, lines 136–145. |  |  |
| **Methods** | | | | | |
| Study Design | 4 | Present key elements of study design early in the paper | Abstract – Methods, lines 31–37.  Introduction lines 138–145.. |  |  |
| Setting | 5 | Describe the setting, locations, and relevant dates, including  periods of recruitment, exposure, follow-up, and data collection | Abstract – Methods, lines 31–37;  Introduction lines 136–145. Methods – Study designed and population. Lines 615-618. – setting and data sources lines 625- 628. |  |  |

| Participants | 6 | (a) *Cohort study* - Give the eligibility criteria, and the sources and methods of selection of participants. Describe methods of follow-up   *Case-control study* - Give the eligibility criteria, and the sources and methods of case ascertainment and control selection. Give the rationale for the choice of cases and controls *Cross-sectional study* - Give the eligibility criteria, and the sources and methods of selection of participants   (b) *Cohort study* - For matched studies, give matching criteria and number of exposed and unexposed   *Case-control study* - For matched studies, give matching criteria and the number of controls per case | Methods – Inclusion and Exclusion Criteria, line 637  Supplementary Figure S1. | RECORD 6.1: The methods of study population selection (such as codes or algorithms used to identify subjects) should be listed in detail. If this is not possible, an explanation should be provided.  RECORD 6.2: Any validation studies of the codes or algorithms used to select the population should be referenced. If validation was conducted for this study and not published elsewhere, detailed methods and results should be provided.  RECORD 6.3: If the study involved linkage of databases, consider use of a flow diagram or other graphical display to demonstrate the data linkage process, including the number of individuals with linked data at each stage. | Methods – Inclusion and Exclusion Criteria lines 637.  Supplementary Figure S1.  Methods – Laboratory Diagnosis and Serotype Determination lines 647.  Supplementary Figure S1 (Study Flow Diagram). |
| --- | --- | --- | --- | --- | --- |
| Variables | 7 | Clearly define all outcomes, exposures, predictors, potential confounders, and effect modifiers. Give diagnostic criteria, if applicable. | Methods – Variables and Definitions line 656 | RECORD 7.1: A complete list of codes and algorithms used to classify exposures, outcomes, confounders, and effect modifiers should be provided. If these cannot be reported, an explanation should be provided. | Methods – Variables and Case Definitions line 656; Supplementary Tables S1 and S2. |
| Data sources/ measurement | 8 | For each variable of interest, give sources of data and details of methods of assessment (measurement).  Describe comparability of assessment methods if there is more than one group | Methods – Laboratory Diagnosis and Serotype Determination lines 640-647 |  |  |

| Bias | 9 | Describe any efforts to address potential sources of bias | Discussion – Limitations lines 575 |  |  |
| --- | --- | --- | --- | --- | --- |
| Study size | 10 | Explain how the study size was arrived at | Methods – Study Population line 618 and Inclusion Criteria line 630; Results, Table 1. |  |  |
| Quantitative variables | 11 | Explain how quantitative variables were handled in the analyses. If applicable, describe  which groupings were chosen, and why | Methods – Statistical Analysis line 671 |  |  |
| Statistical methods | 12 | (a) Describe all statistical methods, including those used to control for confounding (b) Describe any methods used to examine subgroups and interactions (c) Explain how missing data were addressed (d) *Cohort study* - If applicable, explain how loss to follow-up was addressed   *Case-control study* - If applicable, explain how matching of cases and controls was addressed  *Cross-sectional study* - If applicable, describe analytical methods taking account of sampling strategy   (e) Describe any sensitivity analyses | Methods – Statistical Analysis line 671 |  |  |
| Data access and cleaning methods |  | .. | Methods line 628 | RECORD 12.1: Authors should describe the extent to which the investigators had access to the database population used to create the study population. | Methods – Setting and data sources. Lines 628-635 |

|  |  |  |  | RECORD 12.2: Authors should provide information on the data cleaning methods used in the study. | Methods – Setting and data sources lines 628-635 |
| --- | --- | --- | --- | --- | --- |
| Linkage |  | .. |  | RECORD 12.3: State whether the study included person-level, institutional-level, or other data linkage across two or more databases. The methods of linkage and methods of  linkage quality evaluation should be provided. | Not applicable (No linkage between databases was performed) |
| **Results** | | | | | |
| Participants | 13 | (a) Report the numbers of individuals at each stage of the study (*e.g.*, numbers potentially eligible, examined for eligibility, confirmed eligible, included in the study, completing follow-up, and analysed) (b) Give reasons for non-participation at each stage. (c) Consider use of a flow diagram | Results, Table 1 line 152; Supplementary Figure S1. | RECORD 13.1: Describe in detail the selection of the persons included in the study (*i.e.,* study population selection) including filtering based on data quality, data availability and linkage. The selection of included persons can be described in the text and/or by means of the study flow diagram. | Supplementary Figure S1 and  Methods – Inclusion and Exclusion Criteria. |
| Descriptive data | 14 | (a) Give characteristics of study participants (*e.g.*, demographic, clinical, social) and information on exposures and potential confounders (b) Indicate the number of participants with missing data for each variable of interest (c) *Cohort study* - summarise follow-up time (*e.g.*, average and total amount) | Results – Sociodemographic and Clinical Characteristics, Table 1a and Table 1b, line 164. |  |  |
| Outcome data | 15 | *Cohort study* - Report numbers of outcome events or summary measures over time  *Case-control study* - Report numbers in each exposure | Results – Temporal Dynamics and Serotype Circulation, Table 2 and Figures 1–4, lines 204-287. |  |  |

|  |  | category, or summary measures of exposure  *Cross-sectional study* - Report numbers of outcome events or summary measures |  |  |  |
| --- | --- | --- | --- | --- | --- |
| Main results | 16 | (a) Give unadjusted estimates and, if applicable, confounder-adjusted estimates and their precision (e.g., 95% confidence interval). Make clear which confounders were adjusted for and why they were included (b) Report category boundaries when continuous variables were categorized (c) If relevant, consider translating estimates of relative risk into absolute risk for a meaningful time period | Results – Logistic Regression Analyses, Table 3a and Table 3b, lines 333–416 |  |  |
| Other analyses | 17 | Report other analyses done—e.g., analyses of subgroups and interactions, and sensitivity analyses | Results – Spatial Analyses and Serotype-Specific Models line 416, Figures 2–4 and Tables S1–S2 |  |  |
| **Discussion** | | | | | |
| Key results | 18 | Summarise key results with  reference to study objectives | Discussion line 462 |  |  |
| Limitations | 19 | Discuss limitations of the study, taking into account sources of potential bias or imprecision.  Discuss both direction and magnitude of any potential bias | Discussion – Limitations line 575 | RECORD 19.1: Discuss the implications of using data that were not created or collected to answer the specific research question(s). Include discussion of misclassification bias, unmeasured confounding, missing data, and changing eligibility over time, as they pertain to the study being  reported. | Discussion – Limitations and implications of using routinely collected surveillance data line 575 |
| Interpretation | 20 | Give a cautious overall interpretation of results considering objectives, | Discussion – Interpretation of Findings and Conclusions line 598 |  |  |

|  |  | limitations, multiplicity of analyses, results from similar studies, and other relevant evidence |  |  |  |
| --- | --- | --- | --- | --- | --- |
| Generalisability | 21 | Discuss the generalisability (external validity) of the study results | Discussion – Public Health Implications and Generalizability. |  |  |
| **Other Information** | | | | | |
| Funding | 22 | Give the source of funding and the role of the funders for the present study and, if applicable, for the original study on which the present article is based | Funding Statement (Submission system) line 712 |  |  |
| Accessibility of protocol, raw data, and programming  code |  | .. |  | RECORD 22.1: Authors should provide information on how to access any supplemental information such as the study protocol, raw data, or  programming code. | Data Availability Statement and Supplementary Materials. |

*Reference: Benchimol EI, Smeeth L, Guttmann A, Harron K, Moher D, Petersen I, Sørensen HT, von Elm E, Langan SM, the RECORD Working Committee. The REporting of studies Conducted using Observational Routinely-collected health Data (RECORD) Statement. *PLoS Medicine* 2015; in press.

*Checklist is protected under Creative Commons Attribution [(CC BY)](http://creativecommons.org/licenses/by/4.0/) license.
