# Supplementary material for: Early warning of serotype replacement, spatial risk redistribution, and shifting clinical phenotypes: Insights from the 2023–2025 dengue outbreak in Meta, Colombia
Source: PLoS Negl Trop Dis. 2026 Sep 25;20(9):e0014733. doi: 10.1371/journal.pntd.0014733 (PMC13614597; doi:10.1371/journal.pntd.0014733)
Supplement: Supplementary file 2 [file pntd.0014733.s002.docx]

Figure S1. Flowchart of dengue case selection for serotype-specific analyses.

**Figure S1. Flowchart of dengue case selection for serotype-specific analyses.** Of the 3,717 dengue-suspected cases analyzed between January 2023 and September 2025 in the Department of Meta, Colombia, 368 records were excluded due to unavailable serotype information, including 91 cases with NS1 antigen results only, 249 cases with IgM ELISA results only, and 28 records with incomplete laboratory information. The final analytical cohort comprised 3,349 cases with complete virological information and confirmed dengue virus serotyping by RT- PCR, which were included in analyses of temporal trends, serotype circulation, spatial distribution, and clinical characteristics.
