# Supplementary material for: Early warning of serotype replacement, spatial risk redistribution, and shifting clinical phenotypes: Insights from the 2023–2025 dengue outbreak in Meta, Colombia
Source: PLoS Negl Trop Dis. 2026 Sep 25;20(9):e0014733. doi: 10.1371/journal.pntd.0014733 (PMC13614597; doi:10.1371/journal.pntd.0014733)
Supplement: Supplementary file 3 [file pntd.0014733.s003.docx]

| S1 Table. Bivariate analysis of clinical and sociodemographic factors associated with viral positivity | | | | | | | | | |
| --- | --- | --- | --- | --- | --- | --- | --- | --- | --- |
| Associated Factors | | | Positive | | Pearson  Chi-Square | P-value | OR value | 95% Confidence Interval | |
|  | | | No  n (%) | Yes  n (%) |  |  |  | Lower | Upper |
| Temporal | Year | 2023 | 898(50.4) | 884(49.6) | 22.32 | <0.001 | - | - | - |
|  |  | 2024 | 419(57.2) | 314(42.8) |  |  |  |  |  |
|  |  | 2025 | 496(59.5) | 338(40.5) |  |  |  |  |  |
| Sociodemographic | Sex | Female | 759(52.1) | 698(47.9) | 4.33 | 0.037 | 0.86 | 0.75 | 0.99 |
|  |  | Male | 1054(55.7) | 838(44.3) |  |  |  |  |  |
|  | Health insurance type | **Contributory scheme** | 843(56.1) | 661(43.9) | 4.03 | 0.045 | 1.15 | 1 | 1.31 |
|  |  | **Subsidized scheme** | 970(52.6) | 875(47.4) |  |  |  |  |  |
| Clinical characteristics | Fever | **No** | 160(63.5) | 92(36.5) | 9.60 | 0.002 | 1.51 | 1.16 | 1.98 |
|  |  | **Yes** | 1653(53.4) | 1444(46.6) |  |  |  |  |  |
|  | Headache | **No** | 530(60.1) | 352(39.9) | 17.1 | <0.001 | 1.38 | 1.18 | 1.62 |
|  |  | **Yes** | 1283(52) | 1184(48) |  |  |  |  |  |
|  | Retro-orbital pain | **No** | 1040(57.3) | 775(42.7) | 15.9 | <0.001 | 1.32 | 1.15 | 1.51 |
|  |  | **Yes** | 773(50.4) | 761(49.6) |  |  |  |  |  |
|  | Myalgia | **No** | 403(64.6) | 221(35.4) | 33.7 | <0.001 | 1.70 | 1.42 | 2.03 |
|  |  | **Yes** | 1410(51.7) | 1315(48.3) |  |  |  |  |  |
|  | Arthralgia | **No** | 389(61.6) | 243(38.4) | 17.2 | <0.001 | 1.45 | 1.21 | 1.73 |
|  |  | **Yes** | 1424(52.4) | 1293(47.6) |  |  |  |  |  |
|  | Skin rash | **No** | 1543(55.8) | 1221(44.2) | 18.2 | <0.001 | 1.47 | 1.23 | 1.76 |
|  |  | **Yes** | 270(46.2) | 315(53.8) |  |  |  |  |  |
|  | Abdominal pain | **No** | 1339(56.5) | 1031(43.5) | 18.2 | <0.001 | 1.38 | 1.19 | 1.60 |
|  |  | **Yes** | 474(48.4) | 505(51.6) |  |  |  |  |  |
|  | Emesis | **No** | 1482(55.7) | 1179(44.3) | 12.6 | <0.001 | 1.35 | 1.14 | 1.60 |
|  |  | **Yes** | 331(48.1) | 357(51.9) |  |  |  |  |  |
|  | Somnolence  / Irritability | **No** | 1757(53.8) | 1509(46.2) | 6.09 | 0.014 | 0.56 | 0.35 | 0.89 |
|  |  | **Yes** | 56(67.5) | 27(32.5) |  |  |  |  |  |
|  | Hematocrit increase | **No** | 1762(54.6) | 1467(45.4) | 6.78 | 0.009 | 1.62 | 1.12 | 2.34 |
|  |  | **Yes** | 51(42.5) | 69(57.5) |  |  |  |  |  |
|  | Platelet count  <100,000 | **No** | 1397(62.3) | 845(37.7) | 182 | <0.001 | 2.74 | 2.36 | 3.18 |
|  |  | **Yes** | 416(37.6) | 691(62.4) |  |  |  |  |  |
|  | General  hospitalization | **No** | 498(64.3) | 277(35.7) | 41.6 | <0.001 | 1.72 | 1.45 | 2.03 |
|  |  | **Yes** | 1315(51.1) | 1259(48.9) |  |  |  |  |  |
| Geographic  distribution | Acacias | **No** | 1493(53) | 1326(47) | 9.8 | 0.002 | 0.73 | 0.61 | 0.89 |
|  |  | **Yes** | 320(60.4) | 210(39.6) |  |  |  |  |  |
|  | Castilla La Nueva | **No** | 1664(53.4) | 1454(46.6) | 10.7 | 0.001 | 0.63 | 0.47 | 0.83 |
|  |  | **Yes** | 149(64.5) | 82(35.5) |  |  |  |  |  |
|  | Cubarral | **No** | 1802(54.6) | 1501(45.4) | 17.1 | <0.001 | 3.82 | 1.93 | 7.54 |
|  |  | **Yes** | 11(23.9) | 35(76.1) |  |  |  |  |  |
|  | Granada | **No** | 1595(55.9) | 1260(44.1) | 23.36 | <0.001 | 1.60 | 1.32 | 1.94 |
|  |  | **Yes** | 218(44.1) | 276(55.9) |  |  |  |  |  |
|  | Guamal | **No** | 1697(53.5) | 1475(46.5) | 9.78 | 0.002 | 0.60 | 0.44 | 0.83 |
|  |  | **Yes** | 116(65.5) | 61(34.5) |  |  |  |  |  |
|  | Uribe | **No** | 1813(54.2) | 1531(45.8) | 5.91 | 0.015 | 1.00 | 1.00 | 1.00 |
|  |  | **Yes** | 0 | 5(100) |  |  |  |  |  |
|  | Lejanías | **No** | 1732(53.4) | 1511(46.6) | 21.8 | <0.001 | 0.35 | 0.22 | 0.55 |
|  |  | **Yes** | 81(76.4) | 25(23.6) |  |  |  |  |  |
|  | Mesetas | **No** | 1811(54.3) | 1524(45.7) | 8.99 | 0.003 | 7.13 | 1.59 | 31.9 |
|  |  | **Yes** | 2(14.3) | 12(85.7) |  |  |  |  |  |
|  | Puerto Rico | **No** | 1804(54.3) | 1518(45.7) | 4.74 | 0.029 | 2.37 | 1.06 | 5.30 |
|  |  | **Yes** | 9(33.3) | 18(66.7) |  |  |  |  |  |
|  | Villavicencio | **No** | 1373(56.5) | 1057(43.5) | 20 | <0.001 | 1.41 | 1.21 | 1.64 |
|  |  | **Yes** | 440(47.9) | 479(52.1) |  |  |  |  |  |

**S1 Table.** Bivariate analysis of sociodemographic, clinical, and geographic factors associated with dengue virus positivity in the Department of Meta, Colombia (2023–September 2025).
