# Supplementary material for: Early warning of serotype replacement, spatial risk redistribution, and shifting clinical phenotypes: Insights from the 2023–2025 dengue outbreak in Meta, Colombia
Source: PLoS Negl Trop Dis. 2026 Sep 25;20(9):e0014733. doi: 10.1371/journal.pntd.0014733 (PMC13614597; doi:10.1371/journal.pntd.0014733)
Supplement: Supplementary file 4 [file pntd.0014733.s004.docx]

| S2 Table. Bivariate analysis of clinical and sociodemographic factors associated with serotypes in the Department of Meta. | | | | | | | | | |
| --- | --- | --- | --- | --- | --- | --- | --- | --- | --- |
| Associated Factors | | | Positive | | Pearson  Chi-Square | P-value | OR value | 95% Confidence Interval | |
|  |  |  | No  n (%) | Yes  n (%) |  |  |  | Lower | Upper |
| **DENV-1** | | | | | | | | | |
| Temporal | Year | 2023 | 1713 (96.1) | 69(3.9) | 34.9 | <0.001 | - | - | - |
|  |  | 2024 | 717(97.8) | 16(2.2) |  |  |  |  |  |
|  |  | 2025 | 834(100) | 0 |  |  |  |  |  |
| **Clinical factors** | Myalgias | No | 618(99) | 6(1) | 7.70 | 0.006 | 3.07 | 1.33 | 7.08 |
|  |  | Yes | 2646(97.1) | 79(2.9) |  |  |  |  |  |
|  | Severe mucosal bleeding | No | 3180(97.6) | 79(2.4) | 6.37 | 0.012 | 2.87 | 1.21 | 6.78 |
|  |  | Yes | 84(93.3) | 6(6.7) |  |  |  |  |  |
| Geographic  distribution | Acacías | Yes | 500(94.3) | 30(5.7) | 24.81 | <0.001 | 3 | 1.9 | 4.7 |
|  |  | No | 2764(98) | 55(2) |  |  |  |  |  |
|  | Granada | Yes | 473(95.7) | 21(4.3) | 6.87 | 0.009 | 1.9 | 1.1 | 3.2 |
|  |  | No | 2791(97.8) | 64(2.2) |  |  |  |  |  |
|  | Mesetas | Yes | 11(78.6) | 3(21.4) | 20.2 | <0.001 | 10.8 | 2.9 | 39.5 |
|  |  | No | 3253(97.5) | 82(2.5) |  |  |  |  |  |
|  | Puerto López | Yes | 230(99.6) | 1(0.4) | 4.4 | 0.035 | 0.15 | 0.02 | 1.13 |
|  |  | No | 3034(97.3) | 84(2.7) |  |  |  |  |  |
|  | San Martín | Yes | 216(99.5) | 1(0.5) | 4 | 0.04 | 0.16 | 0.02 | 1.21 |
|  |  | No | 3048(97.3) | 84(2.7) |  |  |  |  |  |
| **DENV-2** | | | | | | | | | |
| Temporal | Year | 2023 | 1093(61.3) | 689(38.7) | 295.1 | <0.001 | - | - | - |
|  |  | 2024 | 606(82.7) | 127(17.3) |  |  |  |  |  |
|  |  | 2025 | 758(90.9) | 76(9.1) |  |  |  |  |  |
|  | Headache | No | 693(78.6) | 189(21.4) | 16.6 | <0.001 | 1.46 | 1.21 | 1.75 |
|  |  | Yes | 1764(71.5) | 703(28.5) |  |  |  |  |  |
|  | Myalgias | No | 490(78.5) | 134(21.5) | 10.45 | 0.001 | 1.40 | 1.14 | 1.73 |
|  |  | Yes | 1967(72.2) | 758(27.8) |  |  |  |  |  |
|  | Arthralgia | No | 502(79.4) | 130(20.6) | 14.6 | <0.001 | 1.50 | 1.22 | 1.85 |
|  |  | Yes | 1955(72) | 762(28) |  |  |  |  |  |
|  | Abdominal pain | No | 1771(74.7) | 599(25.3) | 7.68 | 0.006 | 1.26 | 1.07 | 1.49 |
|  |  | Yes | 686(70.1) | 293(29.9) |  |  |  |  |  |
|  | Emesis | No | 1996(75) | 665(25) | 17.9 | <0.001 | 1.47 | 1.23 | 1.77 |
|  |  | Yes | 461(67) | 227(33) |  |  |  |  |  |
|  | Hematocrit increase | No | 2384(73.8) | 845(26.2) | 10 | 0.002 | 1.81 | 1.24 | 2.64 |
|  |  | Yes | 73(60.8) | 47(39.2) |  |  |  |  |  |
|  | Platelet count  <100,000 | No | 1780(79.4) | 462(20.6) | 126 | <0.001 | 2.44 | 2.08 | 2.86 |
|  |  | Yes | 677(61.2) | 430(38.8) |  |  |  |  |  |
|  | hospitalization | No | 624(80.5) | 151(19.5) | 26.3 | <0.001 | 1.67 | 1.37 | 2.03 |
|  |  | Yes | 1833(71.2) | 741(28.8) |  |  |  |  |  |
| Geographic  distribution | Castilla La Nueva | No | 2267(72.7) | 851(27.3) | 10 | 0.002 | 0.57 | 0.40 | 0.81 |
|  |  | Yes | 190(82.3) | 41(17.7) |  |  |  |  |  |
|  | Cubarral | No | 2442(73.9) | 861(26.1) | 39.6 | <0.001 | 5.86 | 3.14 | 10.9 |
|  |  | Yes | 15(32.6) | 31(67.4) |  |  |  |  |  |
|  | Fuente de Oro | No | 2382(73) | 883(27) | 11.1 | <0.001 | 0.32 | 0.16 | 0.64 |
|  |  | Yes | 75(89.3) | 9(10.7) |  |  |  |  |  |
|  | Guamal | No | 2299(72.5) | 873(27.5) | 24.1 | <0.001 | 0.31 | 0.19 | 0.51 |
|  |  | Yes | 158(89.3) | 19(10.7) |  |  |  |  |  |
|  | Puerto Gaitán | No | 2394(73.9) | 845(26.1) | 15 | <0.001 | 2.11 | 1.43 | 3.10 |
|  |  | Yes | 63(57.3) | 47(42.7) |  |  |  |  |  |
|  | Puerto López | No | 2305(73.9) | 813(26.1) | 7.26 | 0.007 | 1.47 | 1.11 | 1.95 |
|  |  | Yes | 152(65.8) | 79(34.2) |  |  |  |  |  |
|  | Puerto Rico | No | 2446(73.6) | 876(26.4) | 14.8 | <0.001 | 4 | 1.87 | 8.78 |
|  |  | Yes | 11(40.7) | 16(59.3) |  |  |  |  |  |
|  | San Carlos de Guaroa | No | 2453(73.5) | 886(26.5) | 5.7 | 0.017 | 4.1 | 1.16 | 14.7 |
|  |  | Yes | 4(40) | 6(60) |  |  |  |  |  |
|  | San Martín | No | 2260(72.2) | 872(27.8) | 36 | <0.001 | 0.26 | 0.16 | 0.42 |
|  |  | Yes | 197(90.8) | 20(9.2) |  |  |  |  |  |
|  | **DENV-3** | | | | | | | | |
| Temporal | Year | 2023 | 1669(93.7) | 113(6.3) | 97.9 | <0.001 | - | - | - |
|  |  | 2024 | 639(87.2) | 94(12.8) |  |  |  |  |  |
|  |  | 2025 | 675(80.9) | 159(19.1) |  |  |  |  |  |
| **Clinical factors** | Health insurance type | **Contributory** | 1361(90.5) | 143(9.5) | 5.66 | 0.017 | 1.30 | 1.04 | 1.63 |
|  |  | **Subsidized** | 1622(87.9) | 223(12.1) |  |  |  |  |  |
|  | Retro-orbital pain | No | 1642(90.5) | 173(9.5) | 7.94 | 0.005 | 1.36 | 1.09 | 1.69 |
|  |  | Yes | 1341(87.4) | 193(12.6) |  |  |  |  |  |
|  | Skin rash | No | 2483(89.8) | 281(10.2) | 9.44 | 0.002 | 1.50 | 1.15 | 1.95 |
|  |  | Yes | 500(85.5) | 85(14.5) |  |  |  |  |  |
|  | Hypothermia | No | 2975(89.2) | 361(10.8) | 10.16 | 0.001 | 5.15 | 1.67 | 15.8 |
|  |  | Yes | 8(61.5) | 5(38.5) |  |  |  |  |  |
|  | Platelet count  <100,000 | No | 2040(91) | 202(9) | 25.6 | <0.001 | 1.75 | 1.41 | 2.18 |
|  |  | Yes | 943(85.2) | 164(14.8) |  |  |  |  |  |
|  | hospitalization | No | 706(91.1) | 69(8.9) | 4.24 | 0.039 | 1.33 | 1.01 | 1.75 |
|  |  | Yes | 2277(88.5) | 297(11.5) |  |  |  |  |  |
| Geographic  distribution | Acacías | No | 2465(87.4) | 354(12.6) | 48.5 | <0.001 | 0.16 | 0.09 | 0.28 |
|  |  | Yes | 518(97.7) | 12(2.3) |  |  |  |  |  |
|  | Cubarral | No | 2937(88.9) | 366(11.1) | 5.72 | 0.017 | 0.985 | 0.980 | 0.989 |
|  |  | Yes | 46(100) | 0 |  |  |  |  |  |
|  | Fuente de Oro | No | 2928(89.7) | 337(10.3) | 49.2 | <0.001 | 4.58 | 2.88 | 7.28 |
|  |  | Yes | 55(65.5) | 29(34.5) |  |  |  |  |  |
|  | Granada | No | 2556(89.5) | 299(10.5) | 4.13 | 0.042 | 1.34 | 1.01 | 1.78 |
|  |  | Yes | 427(86.4) | 67(13.6) |  |  |  |  |  |
|  | Guamal | No | 2809(88.6) | 363(11.4) | 16.3 | <0.001 | 0.13 | 0.04 | 0.42 |
|  |  | Yes | 174(98.3) | 3(1.7) |  |  |  |  |  |
|  | Uribe | No | 2980(89.1) | 364(10.9) | 4.34 | 0.037 | 5.45 | 0.90 | 32.7 |
|  |  | Yes | 3(60) | 2(40) |  |  |  |  |  |
|  | Lejanías | No | 2878(88.7) | 365(11.3) | 11.2 | <0.001 | 0.07 | 0.01 | 0.54 |
|  |  | Yes | 105(99.1) | 1(0.9) |  |  |  |  |  |
|  | Puerto Gaitán | No | 2875(88.8) | 364(11.2) | 9.6 | 0.002 | 0.14 | 0.03 | 0.59 |
|  |  | Yes | 108(98.2) | 2(1.8) |  |  |  |  |  |
|  | Puerto López | No | 2763(88.6) | 355(11.4) | 9.6 | 0.002 | 0.38 | 0.21 | 0.72 |
|  |  | Yes | 220(95.2) | 11(4.8) |  |  |  |  |  |
|  | San Martín | No | 2824(90.2) | 308(9.8) | 59.5 | <0.001 | 3.34 | 2.42 | 4.61 |
|  |  | Yes | 159(73.3) | 58(26.7) |  |  |  |  |  |
|  | Villavicencio | No | 2210(90.9) | 220(9.1) | 31,98 | <0.001 | 1.89 | 1.51 | 2.37 |
|  |  | Yes | 773(84.1) | 146(15.9) |  |  |  |  |  |
| **DENV-4** | | | | | | | | | |
| Temporal | Year | 2023 | 1766(99.1) | 16(0.9) | 172 | <0.001 | - | - | - |
|  |  | 2024 | 656(89.5) | 77(10.5) |  |  |  |  |  |
|  |  | 2025 | 731(87.6) | 103(12.4) |  |  |  |  |  |
| **Clinical factors** | Health insurance type | **Contributory** | 1432(95.2) | 72(4.8) | 5.62 | 0.018 | 1.43 | 1.06 | 1.93 |
|  |  | **Subsidized** | 1721(93.3) | 124(6.7) |  |  |  |  |  |
|  | Myalgias | No | 599(96) | 25(4) | 4.74 | 0.029 | 1.60 | 1.04 | 2.46 |
|  |  | Yes | 2554(93.7) | 171(6.3) |  |  |  |  |  |
| Geographic  distribution | Acacias | No | 2637(93.5) | 182(6.5) | 11.7 | <0.001 | 0.39 | 0.22 | 0.68 |
|  |  | Yes | 516(97.4) | 14(2.6) |  |  |  |  |  |
|  | Cabuyaro | No | 3148(94.3) | 192(5.7) | 24.3 | <0.001 | 13.1 | 3.49 | 49.2 |
|  |  | Yes | 5(55.6) | 4(44.4) |  |  |  |  |  |
|  | El Dorado | No | 3149(94.2) | 194(5.8) | 8.23 | 0.004 | 8.11 | 1.47 | 44.5 |
|  |  | Yes | 4(66.7) | 2(33.3) |  |  |  |  |  |
|  | Granada | No | 2706(94.8) | 149(5.2) | 14.1 | <0.001 | 1.91 | 1.35 | 2.69 |
|  |  | Yes | 447(90.5) | 47(9.5) |  |  |  |  |  |
|  | Guamal | No | 3010(94.9) | 162(5.1) | 60.5 | <0.001 | 4.41 | 2.94 | 6.63 |
|  |  | Yes | 143(80.8) | 34(19.2) |  |  |  |  |  |
|  | Lejanías | No | 3047(94) | 196(6) | 6.8 | 0.009 | 0.966 | 0.960 | 0.973 |
|  |  | Yes | 106(100) | 0 |  |  |  |  |  |
|  | Mesetas | No | 3143(94.2) | 192(5.8) | 13.1 | <0.001 | 6.54 | 2.03 | 21 |
|  |  | Yes | 10(71.4) | 4(28.6) |  |  |  |  |  |
|  | Puerto Gaitán | No | 3043(93.9) | 196(6.1) | 7.07 | 0.008 | 0.96 | 0.95 | 0.97 |
|  |  | Yes | 110(100) | 0 |  |  |  |  |  |
|  | Puerto López | No | 2924(93.8) | 194(6.2) | 11.1 | <0.001 | 0.13 | 0.03 | 0.53 |
|  |  | Yes | 229(99.1) | 2(0.9) |  |  |  |  |  |
|  | Vistahermosa | No | 3114(94.3) | 187(5.7) | 14.7 | <0.001 | 3.84 | 1.83 | 8.05 |
|  |  | Yes | 39(81.3) | 9(18.8) |  |  |  |  |  |

**S2 Table.** Bivariate analysis of sociodemographic, clinical, and geographic factors associated with dengue virus serotypes (DENV-1 to DENV-4) in the Department of Meta, Colombia (2023–September 2025).
