# Supplementary material for: Early warning of serotype replacement, spatial risk redistribution, and shifting clinical phenotypes: Insights from the 2023–2025 dengue outbreak in Meta, Colombia
Source: PLoS Negl Trop Dis. 2026 Sep 25;20(9):e0014733. doi: 10.1371/journal.pntd.0014733 (PMC13614597; doi:10.1371/journal.pntd.0014733)
Supplement: Supplementary file 5 [file pntd.0014733.s006.docx]

Dear Editors and Reviewers,

We sincerely thank the Editor and all reviewers for their careful evaluation of our manuscript and for the constructive comments and suggestions provided. We greatly appreciate the time and expertise dedicated to reviewing our work. We have carefully considered each comment and revised the manuscript accordingly. For ease of review, we provide below a detailed, point-by-point response to all comments raised by the reviewers. All modifications have been incorporated into the revised manuscript. We hope that these revisions satisfactorily address all concerns and that the revised manuscript is now suitable for publication in PLOS Neglected Tropical Diseases.

1. Methods

- Are the objectives of the study clearly articulated with a clear testable hypothesis stated?

- Is the study design appropriate to address the stated objectives?

- Is the population clearly described and appropriate for the hypothesis being tested?

- Is the sample size sufficient to ensure adequate power to address the hypothesis being tested?

- Were correct statistical analysis used to support conclusions?

- Are there concerns about ethical or regulatory requirements being met?

## Reviewer #1:

The major deficiency that should be corrected is a more comprehensive description of the methods used for virus isolation, serotype determination, PCR and NS1 antigen detection.

Response: We clarified in the Methods section that this study was based exclusively on the secondary analysis of routinely collected surveillance data. Laboratory diagnosis results by RT-PCR, NS1 antigen detection, and dengue virus serotype determination were collected and consolidated by the Public Health Laboratory of the Meta Department as part of routine surveillance activities, following the nationally standardized protocols established by the Colombian National Institute of Health (INS). Furthermore, to avoid possible methodological confusion, we specified that RT-PCR was the only technique used for dengue virus serotyping in all cases included in the serotype-specific analyses. Consequently, cases diagnosed solely by NS1 antigen detection or IgM serology, as well as those with incomplete laboratory information, were excluded from the serotype-specific analyses because serotype information was unavailable. The investigators had access only to the results recorded in the surveillance databases and did not perform any laboratory procedures or additional analyses on biological samples. *(This clarification has been incorporated into the Methods section (lines 630–647).*

## Reviewer #2:

The study addresses a clearly relevant and timely question: characterizing the dynamics of serotype replacement, spatial redistribution of dengue transmission risk, and shifts in clinical presentation during the 2023–2025 outbreak in the Department of Meta, Colombia. The objectives are articulated in the Introduction, though a formal testable hypothesis is not explicitly stated.

1. The study design is described inconsistently across sections: the abstract refers to a 'retrospective observational study'; the Methods (p. 5) to an 'analytical, retrospective cross-sectional study'; and the Discussion (p. 22) to a 'retrospective cross-sectional analytical design.'. A cross-sectional design implies a single measurement point. This study analyzes 33 months of continuously collected surveillance data with monthly serotype trend analysis, temporal series modeling, and interannual spatial comparisons — features characteristic of a retrospective longitudinal analysis of routinely collected health data.

The RECORD extension of the STROBE statement (Benchimol et al., J Clin Epidemiol. 2015; 68:16–20) is applicable and should guide the reporting of this manuscript. Authors are requested to: (1) select a single, accurate, and consistent design denomination for all sections; and (2) specify which reporting guideline was used and upload the completed checklist as supplementary material.

Response: We thank the reviewer for this valuable observation. We agree that the term *cross-sectional* was not the most appropriate designation for a study based on 33 months of continuously collected dengue surveillance data, including temporal trend analyses, serotype replacement assessment, and interannual spatial comparisons. Accordingly, we revised the manuscript to ensure a single, accurate, and consistent description of the study design throughout all sections.

The study is now consistently described as a retrospective longitudinal observational study based on routinely collected dengue surveillance data in the Abstract, Introduction, Methods, and Discussion sections.

In addition, following the reviewer’s recommendation, we incorporated the RECORD (Reporting of studies Conducted using Observational Routinely collected health Data) Statement, an extension of the STROBE guideline for studies using routinely collected health data. A statement indicating adherence to the RECORD guideline has been added to the Methods section, and the completed RECORD Checklist has been included as Supplementary File S1. *(This clarification has been incorporated into the Methods section (line 620).)*

Changes made in the manuscript:

- Abstract, Methods section: study design revised.
- Introduction: study design revised.
- Methods: study design revised and RECORD reporting guideline added.
- Discussion: study design terminology standardized.
- Supplementary Material: RECORD Checklist added as Supplementary File S1.

2. The study population (all suspected dengue cases notified in Meta with laboratory testing) is appropriate for the objectives. The use of viral isolation for serotype identification is the diagnostic gold standard and represents a significant methodological strength. The sample size (n = 3,349 virologically confirmed cases) is large and provides adequate power for the primary analyses.

Response: We thank the reviewer for this positive assessment. We agree that the inclusion of all laboratory-tested suspected dengue cases reported in the Department of Meta constitutes an appropriate study population to address the study objectives. We also appreciate the recognition of the large sample size and the use of routinely collected virological surveillance data with available serotype information, which strengthen the robustness of the temporal, spatial, and clinical analyses presented in this study.

Furthermore, to avoid potential methodological confusion, we clarified in the Methods section that RT-PCR was the only technique used for dengue virus serotyping and that serotype determination was performed in all 3,349 samples with complete virological information that were included in the serotype-specific analyses.

No additional changes to the manuscript were required in response to this comment.

3. The text states 'Male sex showed a higher probability of viral positivity compared with females (OR = 0.86; 95% CI: 0.75–0.99).' However, OR = 0.86 (CI entirely below 1.0) indicates a lower probability in males relative to females, not higher. This is a directly verifiable inconsistency within the main manuscript. Authors must verify whether OR = 0.86 is correct (in which case the narrative should state lower probability) or whether the narrative is correct (in which case the OR should be > 1.0) and specify the reference category explicitly in the Methods section.

Response: we thank the reviewer for identifying this inconsistency. We verified the analysis and confirmed that the reported odds ratio (OR = 0.86; 95% CI: 0.75–0.99) was correct; however, the interpretation in the text was inaccurate. The Results section has been revised to indicate that male sex was associated with a lower probability of viral positivity compared with females *(line 297).* In addition, the Methods section now explicitly states that, for binary variables, the first category listed in each contingency table was used as the reference category for odds ratio estimation. These corrections do not affect the statistical results or the conclusions of the study *(line 667).*

4. Unverifiable claim about DENV-4 prevalence (p. 11 vs Table 2). The text states that DENV-4 represented 'approximately 40–50% of serotyped samples in the most recent months' (p. 11). Table 2, however, reports 103 DENV-4 isolates out of 834 total samples in 2025, corresponding to 12.35% of the annual total, or 30.5% of positive isolates (103/338 positives). The 40–50% figure could be accurate for specific months within 2025, but this cannot be verified from Table 2 (annual aggregates) or Figure 1b (100%-stacked bar format that does not allow visual verification). Authors must provide monthly disaggregated data for 2025 serotype distribution to support this claim or reformulate the statement according to verifiable data.

Response: we agree that the statement indicating that DENV-4 represented approximately 40–50% of serotyped samples could not be directly verified from the annual aggregated data presented in Table 2 and Figure 1B. Therefore, the text has been revised. The manuscript now indicates that DENV-4 accounted for 103 isolates in 2025, corresponding to 12.35% of all processed samples and 30.5% of serotype-positive isolates identified that year, which is fully supported by the data presented in Table 2 *(line 216).*

5. No participant flow diagram is provided. The manuscript transitions from n = 3,717 samples analyzed to n = 3,349 confirmed serotyped cases without documenting how many records were excluded by each criterion (inconclusive results; sample-to-collection interval >5 days). This information is required by STROBE item 13.

Response: we have incorporated a flowchart (Supplementary Figure S1) describing the selection process of cases included in the serotype-specific analyses. Of the 3,717 dengue-suspected cases analyzed during the study period, 3,349 had complete virological information and confirmed dengue virus serotyping and were therefore included in the final analyses. A total of 368 records were excluded because serotype information was unavailable, including 91 cases with NS1 antigen results only, 249 cases with IgM ELISA results only, and 28 records with incomplete laboratory information. The Methods section has been revised accordingly, and Supplementary Figure S1 has been added to provide a detailed description of the inclusion and exclusion process *(line 637).*

Reviewer #3:

(No Response)

2. Results


- Does the analysis presented match the analysis plan?

- Are the results clearly and completely presented?

- Are the figures (Tables, Images) of sufficient quality for clarity?

Reviewer #1:

The figures 2-4 are very poor quality and somehow need to be improved to allow reading the text. Results are well presented in the text.

Response: we thank the reviewer for this valuable comment. We have improved the quality and resolution of Figures 2–4 to enhance readability, including clearer municipal labels, legends, and graphical elements. High-resolution versions of these figures have been uploaded as separate files in accordance with the journal's submission requirements. We appreciate the reviewer's positive assessment regarding the presentation of the results in the text.

Reviewer #2:

1. The internal numerical consistency of the primary data tables was independently verified. All annual totals in Table 2 (n = 1,782 in 2023; n = 733 in 2024; n = 834 in 2025; cumulative n = 3,349) are arithmetically correct and internally consistent. Reported percentages are accurate: for example, DENV-2 in 2023 calculates to 686/1,782 = 38.50% (reported as 38.49%). The sums of clinical management categories (775 + 2,011 + 530 + 33 = 3,349) and final clinical classification categories (33 + 1,919 + 1,169 + 228 = 3,349) both coincide exactly with the cohort total, indicating careful data management.

2. Key regression coefficients in Tables 3a and 3b were independently verified. The OR for somnolence/irritability (Table 3a): exp (0.875) = 2.399 (reported as 2.400; IC 95% 1.464–3.931, reported as 1.465–3.930) — correct. The OR per year for DENV-4 (Table 3b): exp (1.011) = 2.748 (reported as 2.748; IC 95% 2.233–3.383, reported as 2.232–3.383) — correct. These verifications confirm the computational accuracy of the main regression outputs.

3. The temporal trend data in Table 2 show a coherent and internally consistent progression of DENV-4: 0.84% of isolates in 2023, 10.50% in 2024, and 12.35% in 2025. This progression supports the narrative of DENV-4 emergence and is consistent with the multivariable model result (OR per year = 2.748). The interannual variation figures for municipalities with data available at both endpoints are also verified: Acacías (2.6% − 9.4% = −6.80%, reported as −6.77%); Villavicencio (12.5% − 17.6% = −5.10%, reported as −5.12%); Granada (11.6% − 7.1% = +4.50%, reported as +4.47%). Minor differences are attributable to rounding.

Response: we thank the reviewer for the detailed verification of the numerical consistency and computational accuracy of the study data. We appreciate the confirmation that the results presented in Tables 2, 3a, and 3b, as well as the temporal and spatial trend analyses, are internally consistent and accurately reported. No changes to the manuscript were necessary in response to these comments.

4. The claim that DENV-4 represented 40–50% of serotyped samples 'in the most recent months' (p. 11) is not verifiable from Table 2 (which reports annual aggregates only) or Figure 1b (100%-stacked format). The 12.35% annual total and 30.5% of 2025 positive isolates are verifiable. The 40–50% figure requires monthly disaggregated data to be confirmed, or the statement should be revised.

Response: we agree that the 40–50% estimate could not be verified from the annual aggregated data presented in the manuscript. Accordingly, the statement has been revised and now reports only values directly supported by Table 2. The text indicates that DENV-4 reached 103 isolates in 2025 and accounted for 30.5% of all serotype-positive cases identified during that year *(line 216).*

5. Regarding figure quality: Figures 2, 3, and 4 are reproduced at insufficient resolution in the PDF, making municipal labels and legend values difficult to read. Figure 4 shows label overlap with proportional circles in high-density municipalities (Villavicencio, Granada). High-resolution versions must be submitted

Response: figures 2, 3, and 4 have been regenerated and submitted as high-resolution TIFF files in accordance with the journal requirements. In addition, Figure 4 was revised to improve readability by adjusting the size and placement of labels and proportional symbols, thereby reducing overlap in municipalities with higher case densities, including Villavicencio and Granada. These modifications improve the clarity of municipal labels, legend values, and the overall visualization of the spatial distribution patterns.

Reviewer #3:

(No Response)

3. Conclusions

- Are the conclusions supported by the data presented?

- Are the limitations of analysis clearly described?

- Do the authors discuss how these data can be helpful to advance our understanding of the topic under study?
- Is public health relevance addressed?

Reviewer #1:

Yes, nice job on conclusions

Response: we sincerely thank the reviewer for this positive assessment of the conclusions section. We appreciate the recognition that the conclusions are well supported by the findings presented in the manuscript.

Reviewer #2:

The central conclusions of the manuscript (progressive DENV-2 to DENV-4 serotype replacement, spatial redistribution of diagnostic positivity toward intermediate municipalities, and emergence of non-classical neurological manifestations as independent predictors of virological positivity) are generally supported by the data presented. The temporal DENV-4 trend in Table 2 is coherent with the multivariable model result. The interannual variation in spatial positivity is documented with verifiable municipal data. The OR for somnolence/irritability (2.40; 95% CI: 1.47–3.93) is correctly calculated and statistically robust. The limitations section is one of the strongest parts of the manuscript. The authors explicitly acknowledge the retrospective design, differential sample distribution across municipalities, absence of genomic characterization of 2025 isolates, and the class-imbalance problem in DENV-4 models. The public health relevance of this manuscript is high and clearly articulated. The documentation of DENV-4 emergence in a region with historically limited DENV-4 circulation, and the identification of non-classical neurological manifestations as emerging clinical predictors in the context of serotype replacement, have direct operational implications for updating dengue clinical screening protocols and for prioritizing laboratory surveillance efforts in intermediate municipalities. These contributions justify publication in PLOS Neglected Tropical Diseases following the required revisions.

Response: we thank the reviewer for these positive comments and for recognizing the methodological rigor, internal consistency, and public health relevance of our findings. We appreciate the reviewer's assessment and have addressed all requested revisions in the revised manuscript.

Reviewer #3:

No Response

**Editorial and Data Presentation Modifications?**

Use this section for editorial suggestions as well as relatively minor modifications of existing data that would enhance clarity. If the only modifications needed are minor and/or editorial, you may wish to recommend “Minor Revision” or “Accept”.

Reviewer #1:

See summary

Reviewer #2:

1. Table 2 caption in Spanish: The descriptive caption for Table 2 (lines 229–231) is written entirely in Spanish: 'Se presentan los recuentos absolutos y porcentajes por serotipo…'. The rest of the manuscript is in English. The caption must be translated to English.

2. Vector insect nomenclature: Aedes aegypti and Aedes albopictus appear in roman (upright) type in the Introduction (p. 4, lines 82–83). According to ICZN rules and PLOS NTD style guidelines, all genus and species names must be italicized at every occurrence throughout the manuscript, including tables and figure legends. On second mention within the same paragraph, the genus may be abbreviated (Ae. aegypti) but must remain italicized.

Response: we thank the reviewer for these helpful editorial observations. The caption of Table 2 has been translated into English to ensure consistency throughout the manuscript. In addition, the scientific names of the mosquito vectors, *Aedes aegypti* and *Aedes albopictus*, have been revised and italicized throughout the manuscript

3. Funding statement contradiction: The submission form states, 'The author(s) received no specific funding for this work,' while the manuscript body states, 'This study was funded by Universidad Cooperativa de Colombia.' These statements are directly contradictory. Both must be corrected to accurately and consistently reflect the funding source.

Response: the funding statements have been corrected and standardized in both the submission form and the manuscript. Both now consistently indicate that this study was funded by Universidad Cooperativa de Colombia.

4. Duplicate references: References 14 and 26 are identical (Katzelnick LC et al. Science. 2017;358(6365):929–32). References 18 and 23 are identical (Salje H et al. Nat Commun. 2021;12(1):1810). Each pair must be consolidated into a single citation.

5. Reference 10-year error: Reference 10 (PAHO 2020 arbovirus bulletin) cites the year 2026 in the URL. This appears to be a typographical error. Please verify and correct.

Response: the reference list was carefully reviewed and corrected accordingly. Duplicate references were identified and consolidated into single citations; specifically, References 14 and 26 (Katzelnick et al., 2017) and References 18 and 23 (Salje et al., 2021) were merged, and all in-text citations and reference numbering were updated accordingly. In addition, Reference 10 was revised, and the year cited in the PAHO arbovirus bulletin URL was confirmed to be a typographical error and corrected in the revised manuscript.

6. Results section structure: The Results section contains 17 numbered subsections, creating structural fragmentation that impedes readability. Consolidation into four to five primary thematic sections is recommended. Section 8 ('Spatiotemporal Dynamics and Multivariable Risk Integration: The DENV-4 Emergence') synthesizes results from earlier sections and is better positioned as an integrative paragraph within the Discussion

Response: to improve readability and reduce structural fragmentation, we reorganized the Results section into five major thematic sections that group related findings and substantially reduce the number of standalone subsections. Specifically, we consolidated the presentation of sociodemographic and clinical characteristics, temporal dynamics and serotype circulation, spatial and spatiotemporal patterns, and factors associated with dengue positivity and serotype distribution. We respectfully retained the former Section 8 as a final integrative results section (currently Section 5). We believe that this section represents a synthesis of quantitatively derived findings rather than an interpretative discussion. It integrates results from the multivariable regression models, spatial analyses, and interannual variation analyses and is accompanied by a summary table (Table 4) that presents original integrated results not shown elsewhere in the manuscript. Similar integrative result sections have been used in epidemiological studies combining spatial and multivariable approaches to identify emerging transmission patterns and geographic risk redistribution. Because this section presents data-driven integration of statistical and spatial findings without comparing our results with previous studies or proposing mechanistic explanations, we consider that it is more appropriately located within the Results section. Furthermore, this integrated presentation contributes to the internal coherence of the manuscript and facilitates the transition toward the Discussion, where the biological, epidemiological, and public health implications of these findings are subsequently interpreted.

Reviewer #3:

I recommend a minor revision for this paper because the main data is strong, but some small changes will make it easier to read and use. Better ways to show data the authors should use a bar chart to show how the virus types changed each year. This would make the rise of DENV 4 very easy to see. A map would also help show the areas where cases went up or down. This would make it clear which towns, like Granada, are now at higher risk. The study found that sleepiness and being annoyed were the best signs of the virus. A simple table should list these signs next to the older signs like head pain or rashes. This would help doctors see that the old signs are not as useful now. Writing and structure the paper uses some hard words that could be simpler. For example, the authors can use the word "movement" instead of the word "redistribution." They should also make sure to say that towns with no data are gaps in the system rather than places with no virus. The findings about DENV 4 are very important for health planning. This info should be at the very start of the paper, so everyone sees the warning right away. Mentioning that this type of virus is new to many people in the area adds more value to the work. Accuracy in numbers The authors should check that the numbers for blood tests and case counts match in every section. They need to stay consistent when they talk about the low levels of blood cells, which is a key sign of the sickness. These small edits will help the paper guide health workers more effectively.

Response: we thank the reviewer for these constructive suggestions and for recognizing the strength and public health relevance of our findings. To improve clarity and readability, we revised the manuscript presentation and structure. The temporal evolution of dengue virus serotypes, including the emergence and progressive increase of DENV-4, is illustrated in Figure 1B. Likewise, Figures 2 and 3 depict the municipal distribution and interannual changes in dengue positivity, highlighting municipalities with increasing transmission, such as Granada.

We also clarified that municipalities without laboratory reports represent surveillance gaps (“silent zones”) rather than areas without viral circulation. To improve readability, we simplified terminology where appropriate while retaining the term “spatial redistribution” in contexts where it accurately describes changes in the geographic concentration of transmission risk.

In addition, to improve the clinical interpretation of our findings, we revised Section 4.3 of the Results to more clearly contrast the associations observed for classical dengue manifestations and emerging neurological manifestations. Specifically, we now indicate that headache, myalgia, and skin rash were inversely associated with virological confirmation, whereas nonspecific neurological manifestations, particularly somnolence and irritability, emerged as the strongest independent clinical predictor of laboratory-confirmed dengue (OR = 2.41; p = 0.001). No additional analyses or tables were incorporated, and the modification was limited to clarifying the interpretation of the existing multivariable results.

The importance of the emergence of DENV-4 and its implications for public health planning have also been further emphasized in the Abstract and Author Summary, including its emergence in a region with historically limited circulation of this serotype. Finally, we conducted a careful review of the manuscript to ensure consistency in case counts, laboratory results, and the reporting of thrombocytopenia throughout all sections of the manuscript.

**Summary and General Comments**

Use this section to provide overall comments, discuss strengths/weaknesses of the study, novelty, significance, general execution and scholarship. You may also include additional comments for the author, including concerns about dual publication, research ethics, or publication ethics. If requesting major revision, please articulate the new experiments that are needed.

Reviewer #1:

1. Comment:

The manuscript by Rodríguez and colleagues on dengue surveillance in Meta, Colombia, constitutes, in my opinion, a very valuable contribution and provides a great deal of interesting information, particularly the description of serotype displacement that occurred over a few years. In general, the manuscript is well written and presented; however, I have several comments that could further enhance its value.

Response: we sincerely thank Reviewer #1 for the positive evaluation of our manuscript and for recognizing the relevance and scientific contribution of our findings. We appreciate the constructive comments and suggestions, which have enabled us to improve the methodological clarity and overall presentation of the study.

2. Comment:

One of the most important aspects of this manuscript is the data on virus isolation and serotyping; however, there is essentially no description of the methods used. Please add to the Methods section a description and references, if possible, for the methods used for virus isolation and serotyping, as well as the methods used for NS1 antigen detection and RT-PCR.

Response: we have clarified the Methods section accordingly. We explicitly state that this study was based exclusively on the secondary analysis of routinely collected surveillance data. Laboratory diagnosis, RT-PCR testing, NS1 antigen detection, and dengue virus serotype determination were not performed by the investigators but were

conducted as part of the routine surveillance activities of the Public Health Laboratory of Meta Department under the protocols established by the Colombian National Institute of Health (INS). The revised manuscript now specifies that the investigators had access only to the laboratory results recorded in the surveillance databases and did not perform any additional laboratory procedures or complementary analyses on biological samples.

3. Comment:

In Figures 2–4, the text on the maps and legends is very difficult or impossible to read. Could this be improved? I understand that this may be difficult, but something needs to be done to improve the quality of these images. Perhaps you could have a larger map with the names of the districts and then eliminate those names on the smaller graphs.

Response: we appreciate this important suggestion and have completely reconstructed Figures 2, 3, and 4 to improve their readability and graphical quality. All main figures have been uploaded as separate high-resolution TIFF files in accordance with the PLOS NTD figure guidelines. Figures 2, 3, and 4 were reconstructed and exported as individual TIFF files, and the final composite figures used in the manuscript were also generated and submitted in TIFF format. In addition, Figures 1, 2, 3, and 4 have been replaced with high-resolution versions (600 dpi) to improve the readability of all labels, legends, and cartographic elements. Figures 2, 3, and 4 were completely reconstructed and re-exported in high resolution to ensure that all components can be clearly examined by editors, reviewers, and readers. Furthermore, Figures 2, 3, and 4 were reconstructed using the official municipal boundary shapefiles provided by the National Administrative Department of Statistics (DANE), Colombia. The maps were prepared de novo in QGIS as original cartographic products, and the corresponding data source and licensing information have been incorporated into the figure legends.

4. Minor comments:

4.1 On lines 60 and 86, you refer to WHO descriptions of dengue: I would use reference [1] for each of these of these statements.

Response: the manuscript has been revised accordingly, and Reference [1], corresponding to the World Health Organization (WHO) dengue guidelines, has been incorporated.

4.2 Table 2: define what you mean by DENV-1/DENV-2 etc. Also, the caption is in Spanish and needs to be translated.

Response: table 2 has been revised to provide explicit definitions of DENV-1, DENV-2, DENV-3, and DENV-4 as dengue virus serotypes 1, 2, 3, and 4, respectively. We also clarified that the combined categories (e.g., DENV-1/DENV-2 and DENV-2/DENV-3) correspond to mixed infections in which two dengue virus serotypes were simultaneously detected in the same sample. In addition, the table caption has been fully translated into English and revised to improve clarity and interpretability.

4.3 Figure 1a: Is the % positive point for 05-2023, correct? It looks too low.

Response: We appreciate this observation and carefully rechecked the original surveillance database and all calculations. The value for May 2023 (05-2023) is correct. Diagnostic positivity was calculated as the proportion of positive samples relative to the total number of samples processed each month. In May 2023, there were 222 positive and 296 negative samples, resulting in a positivity of 42.9%, which was rounded to 43%. Although the absolute number of positive cases remained high during this month, the simultaneous increase in negative samples reduced the proportion of positive results compared with previous months. We carefully reviewed Figure 1a and verified that the plotted values accurately represent the underlying data. To improve interpretability and clarify the calculation of diagnostic positivity, we have revised the figure legend to explicitly state that positivity was calculated as the proportion of positive samples relative to the total number of samples processed each month (positive and negative samples).

4.4 Table 3: I think the numeric values should use periods instead of commas (e.g. 0.190 instead of 0,190). It would also be good to define B, SE, Z and p in the legend of this table for readers not familiar with these analyses.

Response: all decimal separators in Table 3 have been revised to use periods instead of commas, in accordance with international scientific reporting conventions. In addition, the table legends were revised to explicitly define the statistical abbreviations used in the regression models, including B (regression coefficient), SE (standard error), Z (Wald Z statistic), OR (odds ratio), 95% CI (95% confidence interval), and p (probability value). These modifications were made to improve the clarity and interpretability of the results for readers who may not be familiar with these analyses.

Reviewer #2:

Overview. This manuscript reports a comprehensive characterization of the dengue outbreak that occurred in the Department of Meta, Colombia, between January 2023 and September 2025. Using routinely collected surveillance data linked to the Regional Public Health Laboratory database, the authors integrate virological (serotyping by viral isolation), clinical, and geospatial information from 3,349 virologically confirmed cases. The central findings — progressive DENV-2 to DENV-4 serotype replacement, spatial redistribution of diagnostic positivity toward intermediate municipalities, and the emergence of somnolence and irritability as independent predictors of virological confirmation — are original and epidemiologically significant. The manuscript warrants publication in PLOS Neglected Tropical Diseases following major revision.

Strengths. The use of viral isolation for serotype identification — the diagnostic gold standard — is the primary methodological strength. Independent numerical verification confirms the internal consistency of the primary data tables (all annual subtotals in Table 2 are arithmetically correct; clinical classification and management categories in Table 1a sum exactly to n = 3,349) and the accuracy of key regression coefficients (OR for somnolence/irritability: exp(0.875) = 2.399, reported as 2.400; OR per year for DENV-4: exp(1.011) = 2.748, exact match). The temporal coherence of the DENV-4 emergence signal across both raw data (Table 2: 0.84% in 2023 → 10.50% in 2024 → 12.35% in 2025) and the multivariable model (OR = 2.748 per year) strengthens the credibility of this finding. The geospatial identification of surveillance 'silent zones' in six municipalities without 2025 laboratory reports is a valuable operational contribution. The limitations section is transparent and substantive. The manuscript requires revision before acceptance; the authors are asked to address all corrections detailed in the preceding sections of this review.

Response: we thank the reviewer for this thoughtful and encouraging assessment of our work. We appreciate the recognition of the originality and epidemiological relevance of our findings, including the progressive replacement of DENV-2 by DENV-4, the changing spatial patterns of dengue positivity, and the emergence of nonspecific neurological manifestations as independent predictors of virologically confirmed dengue. We also appreciate the reviewer's recognition of the internal consistency of the dataset and the transparency of the limitations section. As suggested, we have carefully addressed all comments and corrections detailed in the subsequent sections of this review and have revised the manuscript accordingly.

Reviewer #3:

(No Response)

Sincerely,

Liliana Marcela Montilla Rodríguez

Corresponding author
