# Supplementary material for: Early warning of serotype replacement, spatial risk redistribution, and shifting clinical phenotypes: Insights from the 2023–2025 dengue outbreak in Meta, Colombia
Source: PLoS Negl Trop Dis. 2026 Sep 25;20(9):e0014733. doi: 10.1371/journal.pntd.0014733 (PMC13614597; doi:10.1371/journal.pntd.0014733)
Supplement: Peer review history

# Peer review: Decision Letter 0

**DOI:** 10.1371/journal.pntd.0014733.r001

**Authors:** Sujatha Sunil, Hassan Hussein

25 May 2026

PNTD-D-26-00241

Early Warning of Serotype Replacement, Spatial Risk Redistribution, and Shifting Clinical Phenotypes: Insights from the 2023–2025 Dengue Outbreak in Meta, Colombia

PLOS Neglected Tropical Diseases

Dear Dr. Montilla Rodriguez,

Thank you for submitting your manuscript to PLOS Neglected Tropical Diseases. After careful consideration, we feel that it has merit but does not fully meet PLOS Neglected Tropical Diseases's publication criteria as it currently stands. Therefore, we invite you to submit a revised version of the manuscript that addresses the points raised during the review process.

Please submit your revised manuscript within by Jul 24 2026 11:59PM. If you will need more time than this to complete your revisions, please reply to this message or contact the journal office at plosntds@plos.org. When you're ready to submit your revision, log on to https://www.editorialmanager.com/pntd/ and select the 'Submissions Needing Revision' folder to locate your manuscript file.

Please include the following items when submitting your revised manuscript:

* A letter that responds to each point raised by the editor and reviewer(s). You should upload this letter as a separate file labeled 'Response to Reviewers'. This file does not need to include responses to any formatting updates and technical items listed in the 'Journal Requirements' section below.

* A marked-up copy of your manuscript that highlights changes made to the original version. You should upload this as a separate file labeled 'Revised Manuscript with Track Changes'.

* An unmarked version of your revised paper without tracked changes. You should upload this as a separate file labeled 'Manuscript'.

If you would like to make changes to your financial disclosure, competing interests statement, or data availability statement, please make these updates within the submission form at the time of resubmission. Guidelines for resubmitting your figure files are available below the reviewer comments at the end of this letter.

As the corresponding author, your ORCID iD is verified in the submission system and will appear in the published article. PLOS supports the use of ORCID, and we encourage all coauthors to register for an ORCID iD and use it as well. Please encourage your coauthors to verify their ORCID iD within the submission system before final acceptance, as unverified ORCID iDs will not appear in the published article. Only the individual author can complete the verification step; PLOS staff cannot verify ORCID iDs on behalf of authors.

We look forward to receiving your revised manuscript.

Kind regards,

Hassan Abdi Hussein

Academic Editor

PLOS Neglected Tropical Diseases

Sujatha Sunil

Section Editor

PLOS Neglected Tropical Diseases

Shaden Kamhawi

co-Editor-in-Chief

PLOS Neglected Tropical Diseases

orcid.org/0000-0003-4304-636XX

Paul Brindley

co-Editor-in-Chief

PLOS Neglected Tropical Diseases

orcid.org/0000-0003-1765-0002

**Journal Requirements:**

If the reviewer comments include a recommendation to cite specific previously published works, please review and evaluate these publications to determine whether they are relevant and should be cited. There is no requirement to cite these works unless the editor has indicated otherwise.

1) Please provide an Author Summary. This should appear in your manuscript between the Abstract (if applicable) and the Introduction, and should be 150-200 words long. The aim should be to make your findings accessible to a wide audience that includes both scientists and non-scientists. Sample summaries can be found on our website under Submission Guidelines:

https://journals.plos.org/plosntds/s/submission-guidelines#loc-parts-of-a-submission

2) Your manuscript's sections are not in the correct order.  Please amend to the following order: Abstract, Introduction, Results, Discussion, and Methods

3) Please upload all main figures as separate Figure files in .tif or .eps format. For more information about how to convert and format your figure files please see our guidelines:

https://journals.plos.org/plosntds/s/figures

4) The resolution of Figures 1, 2, 3, and 4 are very low and somewhat difficult to read. It is important that our Editors and Peer Reviewers are able to read all parts of a submission. Please replace these figures with higher resolution copies.

5) We have noticed that you have uploaded Supporting Information files, but you have not included a list of legends. Please add a full list of legends for your Supporting Information files after the references list.

6) Some material included in your submission may be copyrighted. According to PLOS’s copyright policy, authors who use figures or other material (e.g., graphics, clipart, maps) from another author or copyright holder must demonstrate or obtain permission to publish this material under the Creative Commons Attribution 4.0 International (CC BY 4.0) License used by PLOS journals. Please closely review the details of PLOS’s copyright requirements here: PLOS Licenses and Copyright. If you need to request permissions from a copyright holder, you may use PLOS's Copyright Content Permission form.

Please respond directly to this email and provide any known details concerning your material's license terms and permissions required for reuse, even if you have not yet obtained copyright permissions or are unsure of your material's copyright compatibility. Once you have responded and addressed all other outstanding technical requirements, you may resubmit your manuscript within Editorial Manager.

Potential Copyright Issues:

i) Figures 2, 3, and 4. Please (a) provide a direct link to the base layer of the map (i.e., the country or region border shape) and ensure this is also included in the figure legend; and (b) provide a link to the terms of use / license information for the base layer image or shapefile. We cannot publish proprietary or copyrighted maps (e.g. Google Maps, Mapquest) and the terms of use for your map base layer must be compatible with our CC BY 4.0 license.

Note: if you created the map in a software program like R or ArcGIS, please locate and indicate the source of the basemap shapefile onto which data has been plotted.

If your map was obtained from a copyrighted source please amend the figure so that the base map used is from an openly available source. Alternatively, please provide explicit written permission from the copyright holder granting you the right to publish the material under our CC BY 4.0 license.

If you are unsure whether you can use a map or not, please do reach out and we will be able to help you. The following websites are good examples of where you can source open access or public domain maps:

* U.S. Geological Survey (USGS) - All maps are in the public domain. (http://www.usgs.gov)

* PlaniGlobe - All maps are published under a Creative Commons license so please cite u201cPlaniGlobe, http://www.planiglobe.com, CC BY 2.0u201d in the image credit after the caption. (http://www.planiglobe.com/?lang=enl)

* Natural Earth - All maps are public domain. (http://www.naturalearthdata.com/about/terms-of-use/).

7) In the online submission form, you indicated that your data will be submitted to a repository upon acceptance. We strongly recommend all authors deposit their data before acceptance, as the process can be lengthy and hold up publication timelines. Please note that, though access restrictions are acceptable now, your entire minimal dataset will need to be made freely accessible if your manuscript is accepted for publication. This policy applies to all data except where public deposition would breach compliance with the protocol approved by your research ethics board. If you are unable to adhere to our open data policy, please kindly revise your statement to explain your reasoning and we will seek the editor's input on an exemption.

**Reviewers' Comments:**

Reviewer's Responses to Questions

**Key Review Criteria Required for Acceptance?**

As you describe the new analyses required for acceptance, please consider the following:

**Methods**

-Are the objectives of the study clearly articulated with a clear testable hypothesis stated?

-Is the study design appropriate to address the stated objectives?

-Is the population clearly described and appropriate for the hypothesis being tested?

-Is the sample size sufficient to ensure adequate power to address the hypothesis being tested?

-Were correct statistical analysis used to support conclusions?

-Are there concerns about ethical or regulatory requirements being met?

Reviewer #1: The major deficiency that should be corrected is a more comprehensive description of the methods used for virus isolation, serotype determination, PCR and NS1 antigen detection.

Reviewer #2: The study addresses a clearly relevant and timely question: characterizing the dynamics of serotype replacement, spatial redistribution of dengue transmission risk, and shifts in clinical presentation during the 2023–2025 outbreak in the Department of Meta, Colombia. The objectives are articulated in the Introduction, though a formal testable hypothesis is not explicitly stated.

1. The study design is described inconsistently across sections: the abstract refers to a 'retrospective observational study'; the Methods (p. 5) to an 'analytical, retrospective cross-sectional study'; and the Discussion (p. 22) to a 'retrospective cross-sectional analytical design.'. A cross-sectional design implies a single measurement point. This study analyzes 33 months of continuously collected surveillance data with monthly serotype trend analysis, temporal series modeling, and interannual spatial comparisons — features characteristic of a retrospective longitudinal analysis of routinely collected health data. The RECORD extension of the STROBE statement (Benchimol et al., J Clin Epidemiol. 2015;68:16–20) is applicable and should guide the reporting of this manuscript. Authors are requested to: (1) select a single, accurate, and consistent design denomination for all sections; and (2) specify which reporting guideline was used and upload the completed checklist as supplementary material.

2. The study population (all suspected dengue cases notified in Meta with laboratory testing) is appropriate for the objectives. The use of viral isolation for serotype identification is the diagnostic gold standard and represents a significant methodological strength. The sample size (n = 3,349 virologically confirmed cases) is large and provides adequate power for the primary analyses

3. The text states 'Male sex showed a higher probability of viral positivity compared with females (OR = 0.86; 95% CI: 0.75–0.99).' However, OR = 0.86 (CI entirely below 1.0) indicates a lower probability in males relative to females, not higher. This is a directly verifiable inconsistency within the main manuscript. Authors must verify whether OR = 0.86 is correct (in which case the narrative should state lower probability) or whether the narrative is correct (in which case the OR should be > 1.0), and specify the reference category explicitly in the Methods section.

4. Unverifiable claim about DENV-4 prevalence (p. 11 vs Table 2). The text states that DENV-4 represented 'approximately 40–50% of serotyped samples in the most recent months' (p. 11). Table 2, however, reports 103 DENV-4 isolates out of 834 total samples in 2025, corresponding to 12.35% of the annual total, or 30.5% of positive isolates (103/338 positives). The 40–50% figure could be accurate for specific months within 2025, but this cannot be verified from Table 2 (annual aggregates) or Figure 1b (100%-stacked bar format that does not allow visual verification). Authors must provide monthly disaggregated data for 2025 serotype distribution to support this claim, or reformulate the statement according to verifiable data.

No participant flow diagram is provided. The manuscript transitions from n = 3,717 samples analyzed to n = 3,349 confirmed serotyped cases without documenting how many records were excluded by each criterion (inconclusive results; sample-to-collection interval >5 days). This information is required by STROBE item 13.

Reviewer #3: (No Response)

**********

**Results**

-Does the analysis presented match the analysis plan?

-Are the results clearly and completely presented?

-Are the figures (Tables, Images) of sufficient quality for clarity?

Reviewer #1: The figures 2-4 are very poor quality and somehow need to be improved to allow reading the text. Results are well presented in the text.

Reviewer #2: The internal numerical consistency of the primary data tables was independently verified. All annual totals in Table 2 (n = 1,782 in 2023; n = 733 in 2024; n = 834 in 2025; cumulative n = 3,349) are arithmetically correct and internally consistent. Reported percentages are accurate: for example, DENV-2 in 2023 calculates to 686/1,782 = 38.50% (reported as 38.49%). The sums of clinical management categories (775 + 2,011 + 530 + 33 = 3,349) and final clinical classification categories (33 + 1,919 + 1,169 + 228 = 3,349) both coincide exactly with the cohort total, indicating careful data management.

2. Key regression coefficients in Tables 3a and 3b were independently verified. The OR for somnolence/irritability (Table 3a): exp(0.875) = 2.399 (reported as 2.400; IC 95% 1.464–3.931, reported as 1.465–3.930) — correct. The OR per year for DENV-4 (Table 3b): exp(1.011) = 2.748 (reported as 2.748; IC 95% 2.233–3.383, reported as 2.232–3.383) — correct. These verifications confirm the computational accuracy of the main regression outputs.

3. The temporal trend data in Table 2 show a coherent and internally consistent progression of DENV-4: 0.84% of isolates in 2023, 10.50% in 2024, and 12.35% in 2025. This progression supports the narrative of DENV-4 emergence and is consistent with the multivariable model result (OR per year = 2.748). The interannual variation figures for municipalities with data available at both endpoints are also verified: Acacías (2.6% − 9.4% = −6.80%, reported as −6.77%); Villavicencio (12.5% − 17.6% = −5.10%, reported as −5.12%); Granada (11.6% − 7.1% = +4.50%, reported as +4.47%). Minor differences are attributable to rounding.

4. The claim that DENV-4 represented 40–50% of serotyped samples 'in the most recent months' (p. 11) is not verifiable from Table 2 (which reports annual aggregates only) or Figure 1b (100%-stacked format). The 12.35% annual total and 30.5% of 2025 positive isolates are verifiable. The 40–50% figure requires monthly disaggregated data to be confirmed or the statement should be revised.

5. Regarding figure quality: Figures 2, 3, and 4 are reproduced at insufficient resolution in the PDF, making municipal labels and legend values difficult to read. Figure 4 shows label overlap with proportional circles in high-density municipalities (Villavicencio, Granada). High-resolution versions must be submitted

Reviewer #3: (No Response)

**********

**Conclusions**

-Are the conclusions supported by the data presented?

-Are the limitations of analysis clearly described?

-Do the authors discuss how these data can be helpful to advance our understanding of the topic under study?

-Is public health relevance addressed?

Reviewer #1: Yes, nice job on conclusions

Reviewer #2: The central conclusions of the manuscript (progressive DENV-2 to DENV-4 serotype replacement, spatial redistribution of diagnostic positivity toward intermediate municipalities, and emergence of non-classical neurological manifestations as independent predictors of virological positivity) are generally supported by the data presented. The temporal DENV-4 trend in Table 2 is coherent with the multivariable model result. The interannual variation in spatial positivity is documented with verifiable municipal data. The OR for somnolence/irritability (2.40; 95% CI: 1.47–3.93) is correctly calculated and statistically robust.

1. The limitations section is one of the strongest parts of the manuscript. The authors explicitly acknowledge the retrospective design, differential sample distribution across municipalities, absence of genomic characterization of 2025 isolates, and the class-imbalance problem in DENV-4 models.

2. The public health relevance of this manuscript is high and clearly articulated. The documentation of DENV-4 emergence in a region with historically limited DENV-4 circulation, and the identification of non-classical neurological manifestations as emerging clinical predictors in the context of serotype replacement, have direct operational implications for updating dengue clinical screening protocols and for prioritizing laboratory surveillance efforts in intermediate municipalities. These contributions justify publication in PLOS Neglected Tropical Diseases following the required revisions.

Reviewer #3: (No Response)

**********

**Editorial and Data Presentation Modifications?**

Use this section for editorial suggestions as well as relatively minor modifications of existing data that would enhance clarity. If the only modifications needed are minor and/or editorial, you may wish to recommend “Minor Revision” or “Accept”.

Reviewer #1: See summary

Reviewer #2: 1. Table 2 caption in Spanish: The descriptive caption for Table 2 (lines 229–231) is written entirely in Spanish: 'Se presentan los recuentos absolutos y porcentajes por serotipo…'. The rest of the manuscript is in English. The caption must be translated to English.

2. Vector insect nomenclature: Aedes aegypti and Aedes albopictus appear in roman (upright) type in the Introduction (p. 4, lines 82–83). According to ICZN rules and PLOS NTD style guidelines, all genus and species names must be italicized at every occurrence throughout the manuscript, including tables and figure legends. On second mention within the same paragraph, the genus may be abbreviated (Ae. aegypti) but must remain italicized.

3. Funding statement contradiction: The submission form states 'The author(s) received no specific funding for this work,' while the manuscript body states 'This study was funded by Universidad Cooperativa de Colombia.' These statements are directly contradictory. Both must be corrected to accurately and consistently reflect the funding source.

4. Duplicate references: References 14 and 26 are identical (Katzelnick LC et al. Science. 2017;358(6365):929–32). References 18 and 23 are identical (Salje H et al. Nat Commun. 2021;12(1):1810). Each pair must be consolidated into a single citation.

5. Reference 10 year error: Reference 10 (PAHO 2020 arbovirus bulletin) cites the year 2026 in the URL. This appears to be a typographical error. Please verify and correct.

6. Results section structure: The Results section contains 17 numbered subsections, creating structural fragmentation that impedes readability. Consolidation into four to five primary thematic sections is recommended. Section 8 ('Spatiotemporal Dynamics and Multivariable Risk Integration: The DENV-4 Emergence') synthesizes results from earlier sections and is better positioned as an integrative paragraph within the Discussion

Reviewer #3: I recommend a minor revision for this paper because the main data is strong, but some small changes will make it easier to read and use. Better ways to show data The authors should use a bar chart to show how the virus types changed each year. This would make the rise of DENV 4 very easy to see. A map would also help show the areas where cases went up or down. This would make it clear which towns, like Granada, are now at higher risk. The study found that sleepiness and being annoyed were the best signs of the virus. A simple table should list these signs next to the older signs like head pain or rashes. This would help doctors see that the old signs are not as useful now. Writing and structure The paper uses some hard words that could be simpler. For example, the authors can use the word "movement" instead of the word "redistribution." They should also make sure to say that towns with no data are gaps in the system rather than places with no virus. The findings about DENV 4 are very important for health planning. This info should be at the very start of the paper so everyone sees the warning right away. Mentioning that this type of virus is new to many people in the area adds more value to the work. Accuracy in numbers The authors should check that the numbers for blood tests and case counts match in every section. They need to stay consistent when they talk about the low levels of blood cells, which is a key sign of the sickness. These small edits will help the paper guide health workers more effectively.

**********

**Summary and General Comments**

Use this section to provide overall comments, discuss strengths/weaknesses of the study, novelty, significance, general execution and scholarship. You may also include additional comments for the author, including concerns about dual publication, research ethics, or publication ethics. If requesting major revision, please articulate the new experiments that are needed.

Reviewer #1: The manuscript by Rodríguez and colleagues on dengue surveillance in Meta, Columbia is what I consider quite a valuable contribution and provides a great deal of interesting information, especially the description of serotype displacement that occurred over a few years. The paper is generally quite well written and presented, but I have several comments to enhance the value of this manuscript.

One of the important aspects of this manuscript is the data on virus isolation and serotyping, but there is essentially no description of the methods used. Please add to the methods section a description and references if possible for methods used for virus isolation and serotyping plus the methods used for NS1 antigen detection (a kit I assume) and rtPCR.

In Figures 2-4 the text on the maps and legends is very difficult or impossible to read. Can this be improved? I know this might be difficult, but something needs to be done to improve the quality of these images. Perhaps you could have a larger map with the names of the districts and then eliminate those names on the smaller graphs?

Minor comments:

- On lines 60 and 86, you refer to WHO descriptions of dengue: I would use reference [1] for each of these of these statements.

- Table 2: define what you mean by DENV-1/DENV-2 etc. Also, the caption is in Spanish and needs to be translated.

- Figure 1a: Is the % positive point for 05-2023 correct? It looks too low.

- Table 3: I think the numeric values should use periods instead of commas (e.g. 0.190 instead of 0,190). It would also be good to define B, SE, Z and p in the legend of this table for readers not familiar with these analyses.

Reviewer #2: Overview. This manuscript reports a comprehensive characterization of the dengue outbreak that occurred in the Department of Meta, Colombia, between January 2023 and September 2025. Using routinely collected surveillance data linked to the Regional Public Health Laboratory database, the authors integrate virological (serotyping by viral isolation), clinical, and geospatial information from 3,349 virologically confirmed cases. The central findings — progressive DENV-2 to DENV-4 serotype replacement, spatial redistribution of diagnostic positivity toward intermediate municipalities, and the emergence of somnolence and irritability as independent predictors of virological confirmation — are original and epidemiologically significant. The manuscript warrants publication in PLOS Neglected Tropical Diseases following major revision.

Strengths. The use of viral isolation for serotype identification — the diagnostic gold standard — is the primary methodological strength. Independent numerical verification confirms the internal consistency of the primary data tables (all annual subtotals in Table 2 are arithmetically correct; clinical classification and management categories in Table 1a sum exactly to n = 3,349) and the accuracy of key regression coefficients (OR for somnolence/irritability: exp(0.875) = 2.399, reported as 2.400; OR per year for DENV-4: exp(1.011) = 2.748, exact match). The temporal coherence of the DENV-4 emergence signal across both raw data (Table 2: 0.84% in 2023 → 10.50% in 2024 → 12.35% in 2025) and the multivariable model (OR = 2.748 per year) strengthens the credibility of this finding. The geospatial identification of surveillance 'silent zones' in six municipalities without 2025 laboratory reports is a valuable operational contribution. The limitations section is transparent and substantive.

The manuscript requires revision before acceptance; the authors are asked to address all corrections detailed in the preceding sections of this review.

Reviewer #3: (No Response)

**********

PLOS authors have the option to publish the peer review history of their article (what does this mean?). If published, this will include your full peer review and any attached files.

If you choose “no”, your identity will remain anonymous but your review may still be made public.

**Do you want your identity to be public for this peer review?**  For information about this choice, including consent withdrawal, please see our Privacy Policy.

Reviewer #1: No

Reviewer #2: No

Reviewer #3: No

**Figure resubmission:**

While revising your submission, we strongly recommend that you use PLOS’s NAAS tool (https://ngplosjournals.pagemajik.ai/artanalysis) to test your figure files. NAAS can convert your figure files to the TIFF file type and meet basic requirements (such as print size, resolution), or provide you with a report on issues that do not meet our requirements and that NAAS cannot fix.

**Reproducibility:**

To enhance the reproducibility of your results, we recommend that authors of applicable studies deposit laboratory protocols in protocols.io, where a protocol can be assigned its own identifier (DOI) such that it can be cited independently in the future. Additionally, PLOS ONE offers an option to publish peer-reviewed clinical study protocols. Read more information on sharing protocols at https://plos.org/protocols?utm_medium=editorial-email&utm_source=authorletters&utm_campaign=protocols

# Author comment: Author response to Decision Letter 1

**DOI:** 10.1371/journal.pntd.0014733.r002

24 Jun 2026

Attachment Submitted filename: Response to Reviewers.docx

# Peer review: Decision Letter 1

**DOI:** 10.1371/journal.pntd.0014733.r003

**Authors:** Sujatha Sunil, Hassan Hussein

7 Sep 2026

Dear Dra Montilla Rodriguez,

We are pleased to inform you that your manuscript 'Early Warning of Serotype Replacement, Spatial Risk Redistribution, and Shifting Clinical Phenotypes: Insights from the 2023–2025 Dengue Outbreak in Meta, Colombia' has been provisionally accepted for publication in PLOS Neglected Tropical Diseases.

Before your manuscript can be formally accepted you will need to complete some formatting changes, which you will receive in a follow up email. A member of our team will be in touch with a set of requests.

Please note that your manuscript will not be scheduled for publication until you have made the required changes, so a swift response is appreciated.

IMPORTANT: The editorial review process is now complete. PLOS will only permit corrections to spelling, formatting or significant scientific errors from this point onwards. Requests for major changes, or any which affect the scientific understanding of your work, will cause delays to the publication date of your manuscript.

Should you, your institution's press office or the journal office choose to press release your paper, you will automatically be opted out of early publication. We ask that you notify us now if you or your institution is planning to press release the article. All press must be co-ordinated with PLOS.

Thank you again for supporting Open Access publishing; we are looking forward to publishing your work in PLOS Neglected Tropical Diseases.

Best regards,

Hassan Abdi Hussein

Academic Editor

PLOS Neglected Tropical Diseases

Sujatha Sunil

Section Editor

PLOS Neglected Tropical Diseases

Shaden Kamhawi

co-Editor-in-Chief

PLOS Neglected Tropical Diseases

orcid.org/0000-0003-4304-636XX

Paul Brindley

co-Editor-in-Chief

PLOS Neglected Tropical Diseases

orcid.org/0000-0003-1765-0002

***********************************************************

p.p1 {margin: 0.0px 0.0px 0.0px 0.0px; line-height: 16.0px; font: 14.0px Arial; color: #323333; -webkit-text-stroke: #323333}span.s1 {font-kerning: none

Reviewer's Responses to Questions

**Key Review Criteria Required for Acceptance?**

As you describe the new analyses required for acceptance, please consider the following:

**Methods**

-Are the objectives of the study clearly articulated with a clear testable hypothesis stated?

-Is the study design appropriate to address the stated objectives?

-Is the population clearly described and appropriate for the hypothesis being tested?

-Is the sample size sufficient to ensure adequate power to address the hypothesis being tested?

-Were correct statistical analysis used to support conclusions?

-Are there concerns about ethical or regulatory requirements being met?

Reviewer #1: The authors have clarified that these are retrospective data and the analyses were conducted by other laboratories.

Reviewer #2: (No Response)

**********

**Results**

-Does the analysis presented match the analysis plan?

-Are the results clearly and completely presented?

-Are the figures (Tables, Images) of sufficient quality for clarity?

Reviewer #1: Revisions acceptable.

Reviewer #2: (No Response)

**********

**Conclusions**

-Are the conclusions supported by the data presented?

-Are the limitations of analysis clearly described?

-Do the authors discuss how these data can be helpful to advance our understanding of the topic under study?

-Is public health relevance addressed?

Reviewer #1: Conclusions appear valid and match the results presented.

Reviewer #2: (No Response)

**********

**Editorial and Data Presentation Modifications?**

Use this section for editorial suggestions as well as relatively minor modifications of existing data that would enhance clarity. If the only modifications needed are minor and/or editorial, you may wish to recommend “Minor Revision” or “Accept”.

Reviewer #1: No comments

Reviewer #2: (No Response)

**********

**Summary and General Comments**

Use this section to provide overall comments, discuss strengths/weaknesses of the study, novelty, significance, general execution and scholarship. You may also include additional comments for the author, including concerns about dual publication, research ethics, or publication ethics. If requesting major revision, please articulate the new experiments that are needed.

Reviewer #1: THe authors have responded well to reviewer comments and the manuscript appears to be improved and a valuable contribution.

Reviewer #2: (No Response)

**********

PLOS authors have the option to publish the peer review history of their article (what does this mean?). If published, this will include your full peer review and any attached files.

If you choose “no”, your identity will remain anonymous but your review may still be made public.

**Do you want your identity to be public for this peer review?**  For information about this choice, including consent withdrawal, please see our Privacy Policy.

Reviewer #1: No

Reviewer #2: No

# Editor report: Acceptance letter

**DOI:** 10.1371/journal.pntd.0014733.r004

**Authors:** Sujatha Sunil, Hassan Hussein

Dear Dra Montilla Rodriguez,

We are delighted to inform you that your manuscript, "Early Warning of Serotype Replacement, Spatial Risk Redistribution, and Shifting Clinical Phenotypes: Insights from the 2023–2025 Dengue Outbreak in Meta, Colombia," has been formally accepted for publication in PLOS Neglected Tropical Diseases.

We have now passed your article onto the PLOS Production Department who will complete the rest of the publication process. All authors will receive a confirmation email upon publication.

The corresponding author will soon be receiving a typeset proof for review, to ensure errors have not been introduced during production. Please review the PDF proof of your manuscript carefully, as this is the last chance to correct any scientific or type-setting errors. Please note that major changes, or those which affect the scientific understanding of the work, will likely cause delays to the publication date of your manuscript. Note: Proofs for Front Matter articles (Editorial, Viewpoint, Symposium, Review, etc...) are generated on a different schedule and may not be made available as quickly.

Soon after your final files are uploaded, the early version of your manuscript will be published online unless you opted out of this process. The date of the early version will be your article's publication date. The final article will be published to the same URL, and all versions of the paper will be accessible to readers.

For Research Articles, you will receive an invoice from PLOS for your publication fee after your manuscript has reached the completed accept phase. If you receive an email requesting payment before acceptance or for any other service, this may be a phishing scheme. Learn how to identify phishing emails and protect your accounts at https://explore.plos.org/phishing.

Thank you again for supporting open-access publishing; we are looking forward to publishing your work in PLOS Neglected Tropical Diseases.

Best regards,

Shaden Kamhawi

co-Editor-in-Chief

PLOS Neglected Tropical Diseases

Paul Brindley

co-Editor-in-Chief

PLOS Neglected Tropical Diseases
